# Agent-Based Model of Therapeutic Adipose-Derived Stromal Cell Trafficking during Ischemia Predicts Ability To Roll on P-Selectin

**DOI:** 10.1371/journal.pcbi.1000294

**Published:** 2009-02-27

**Authors:** Alexander M. Bailey, Michael B. Lawrence, Hulan Shang, Adam J. Katz, Shayn M. Peirce

**Affiliations:** 1 Department of Biomedical Engineering, University of Virginia, Charlottesville, Virginia, United States of America; 2 Department of Plastic Surgery, University of Virginia, Charlottesville, Virginia, United States of America; University of California San Francisco, United States of America

## Abstract

Intravenous delivery of human adipose-derived stromal cells (hASCs) is a promising option for the treatment of ischemia. After delivery, hASCs that reside and persist in the injured extravascular space have been shown to aid recovery of tissue perfusion and function, although low rates of incorporation currently limit the safety and efficacy of these therapies. We submit that a better understanding of the trafficking of therapeutic hASCs through the microcirculation is needed to address this and that selective control over their homing (organ- and injury-specific) may be possible by targeting bottlenecks in the homing process. This process, however, is incredibly complex, which merited the use of computational techniques to speed the rate of discovery. We developed a multicell agent-based model (ABM) of hASC trafficking during acute skeletal muscle ischemia, based on over 150 literature-based rules instituted in Netlogo and MatLab software programs. *In silico*, trafficking phenomena within cell populations emerged as a result of the dynamic interactions between adhesion molecule expression, chemokine secretion, integrin affinity states, hemodynamics and microvascular network architectures. As verification, the model reasonably reproduced key aspects of ischemia and trafficking behavior including increases in wall shear stress, upregulation of key cellular adhesion molecules expressed on injured endothelium, increased secretion of inflammatory chemokines and cytokines, quantified levels of monocyte extravasation in selectin knockouts, and circulating monocyte rolling distances. Successful ABM verification prompted us to conduct a series of systematic knockouts *in silico* aimed at identifying the most critical parameters mediating hASC trafficking. Simulations predicted the necessity of an unknown selectin-binding molecule to achieve hASC extravasation, in addition to any rolling behavior mediated by hASC surface expression of CD15s, CD34, CD62e, CD62p, or CD65. *In vitro* experiments confirmed this prediction; a subpopulation of hASCs slowly rolled on immobilized P-selectin at speeds as low as 2 µm/s. Thus, our work led to a fundamentally new understanding of hASC biology, which may have important therapeutic implications.

## Author Summary

Ischemic pathologies, such as acute myocardial infarction and peripheral vascular disease, continue to be associated with high morbidities and mortalities. Recently, therapies wherein adult stem cells are injected into the circulation have been shown to increase blood flow and help to restore tissue function following injury. Pre-clinical animal models and human trials have shown successes utilizing this approach, but variable trafficking efficiencies and low incorporation of cells into the injured tissue severely limit effectiveness and may preclude clinical adoption. To address this, we sought to study the complex process of how injected stem cells traffic through the microcirculation and home to sites of injury, in an effort to identify bottlenecks in this process that could be manipulated for therapeutic gain. We developed an agent-based computer model to speed the rate of discovery, and we identified a key cell–cell adhesion interaction that could be targeted to enhance stem cell homing efficiencies during injectable stem cell therapies.

## Introduction

Intravenous (i.v.) delivery of therapeutic stem cells is a promising option for the treatment of ischemic injuries. Therapeutic cells that reside and persist (i.e., incorporate) in the injured extravascular space have been shown to aid recovery of tissue perfusion and function, although low rates of incorporation currently limit the safety and efficacy of these therapies [1]. It follows that methods to increase the number of incorporated cells may lead to better clinical outcomes. To achieve this, we submit that a better understanding of stem cell trafficking through the microvasculature prior to incorporation is necessary and that computational modeling techniques could speed the rate of discovery; current investigations however are hampered by the processes' inherent complexity. Towards this end, we present an agent-based computational model (ABM) of therapeutic stem cell trafficking during skeletal muscle ischemia that is capable of generating new hypotheses quickly and cost-effectively.

**Figure 1:**
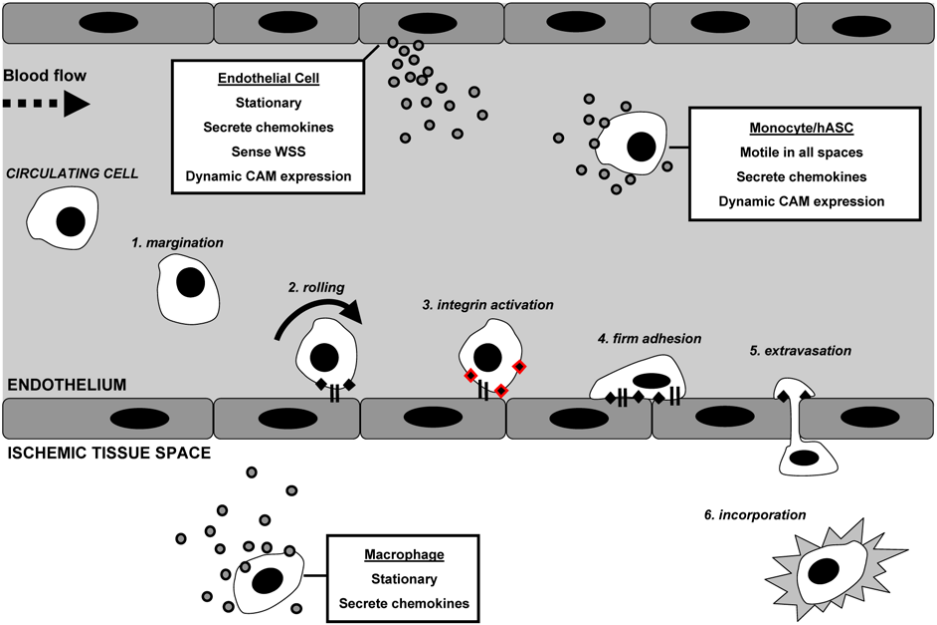
Graphical representation of the adhesion cascade during acute skeletal muscle ischemia, as instituted in the ABM. The adhesion cascade generally proceeds sequentially before incorporation, and is mediated by the interplay between adhesion molecule expression, chemokines and cytokines, hemodynamics, and survey behavior. Interactions are complex spatially and temporally, and cell phenotypes accounted for within simulations are tissue macrophages, endothelial cells making up the blood vessel wall, circulating monocytes, and circulating human adipose-derived stromal cells.

The process by which endogenous circulating cells traffic to sites of general injury and gain access to the extravascular tissue space is referred to as the adhesion cascade [2]. Most often studied in leukocyte subpopulations (e.g., neutrophils and monocytes), it is applicable to the mobilization of circulating stem cells to sites of injury [3]. Depicted in Figure 1, the adhesion cascade consists of a series of sequential events including margination, rolling, integrin activation, firm adhesion, and trans-endothelial migration (extravasation). This complex process is mediated by the interplay between cellular adhesion molecule (CAM) expression, chemokines and cytokines, and hemodynamics [4]–[14].

During skeletal muscle ischemia, tissue damage and dysfunction results from decreases in a tissue's blood supply if nutrient and oxygen delivery cannot meet metabolic demand. As the blood supply decreases, reductions in blood volume flow rates through the downstream vasculature lead to reduced wall shear stresses (WSS), blood flow velocities, and hydrostatic pressures. Conversely, arterial and collateral vessels up-stream of the ligation experience increases in blood volume flow rates, as blood flow is re-routed through intact vasculature (causing vessel swelling and increases in WSS and hydrostatic pressures) [15].

Altered blood flow profiles activate endothelial and perivascular cells through changes in WSS and circumferential stress, respectively, which initiate a complex cascade of events to mobilize circulating cells to the site of injury. Activated endothelial cells (ECs) increase their surface expression of key CAMs, including the selectins (e.g., E-selectin and P-selectin) to support rolling, and the integrins to support firm adhesion and extravasation of circulating cells to the endothelium [15]. The major integrin receptors preferentially expressed on ischemic endothelium include vascular cell adhesion molecule-1 (VCAM-1) and intracellular adhesion molecule-1 (ICAM-1). In concert, activated ECs and perivascular cells also secrete numerous growth factors, chemokines, and cytokines, such as monocyte chemoattractant protein-1 (MCP-1), tumor necrosis factor-α (TNF-α), and interleukin-1β (IL-1β) to increase binding affinities and ensure a higher probability of circulating cell adhesion to the endothelium [16]. Through these mechanisms and others, circulating cells are able to home to ischemic injury sites, adhere to the endothelium, extravasate, and incorporate into the injured tissue.

This paper focuses on therapeutically delivered stem cell populations, or cells that are injected i.v. in order to repair or regenerate injured tissues. It has been hypothesized that i.v.-injected stem cells adhere to activated endothelium using mechanisms that are similar to those of endogenous leukocytes. Moreover, it has been proposed that these adhesive interactions are rate-limiting for an effective therapeutic response [1]. We have chosen to investigate hASCs primarily for their widespread availability and potential clinical impact. Similar to other stem cell populations, hASCs are characterized by a high proliferation and differentiation potential [17] and express many known stem cell-associated markers [18],[19]. They are isolated from adipose tissue following routine intra-operative suction lipectomy or panniculectomy procedures and have shown effectiveness in restoring perfusion to ischemic tissue following i.v. delivery [20],[21]. hASC homing and trafficking capabilities [22], however, are relatively under-studied, and this paper presents a novel computational platform for investigation into this important, but currently ambiguous research area.

We utilized ABM techniques to interrogate therapeutic hASC trafficking during ischemia. Adopting an agent-oriented approach to study the stem cell-specific adhesion cascade has been proposed previously by us [23] and others [24],[25]. Here, we expanded the scope of our prior ABM of monocyte trafficking in healthy skeletal muscle microvasculature to include therapeutically delivered stem cells, tissue-resident macrophages, circulating monocytes, endothelial cells, ischemic injury, and a larger microvascular network. The adhesion cascade was simplified to three primary parameters: CAM expression, hemodynamic forces, and chemokine and cytokine secretion and exposure. A network blood flow analysis program [26], instituted in MATLAB, calculated pressure, flow velocities, and wall shear stresses within the simulated microvascular network, while the other parameters were governed in the Netlogo software program [27] by over 150 rules derived from independent peer-reviewed literature (Text S1).

Model verification was performed by comparing simulation results to data from independent peer-reviewed literature including: (1) monocytes' ability to extravasate independent of selectin-mediated rolling; (2) the preferential upregulation of endothelial CAMs implicated in circulating cell adhesion; (3) the increased secretion of inflammatory chemokines and cytokines by ischemic endothelium; (4) increased WSS and network flow rates in collateral microvascular networks; and (5) rolling distances of circulating monocytes.

Following model verification, the i.v. delivery of therapeutic hASCs was simulated. Early simulations showed unexpected levels of hASC extravasation, which prompted a re-examination of the rule-set and the formulation and testing of a new hypothesis. Rolling via the selectins has been shown to be critical for the homing of other circulating cell populations and is the rate-limiting step during neutrophil trafficking [2],[28]. hASCs do not possess the dominant ligand for the selectins (P-selectin glycoprotein ligand-1 (PSGL-1)), but we hypothesized that they were able to roll on selectins independent of PSGL-1 expression and that this may be a rate-limiting step, as well. Systematic knockout experiments *in silico* supported this hypothesis, and we subsequently validated our model predictions *in vitro* in parallel plate flow chamber assays. In this way, the model was used as a tool and led to new understandings after it behaved unexpectedly. Furthermore, the work suggests that selectin interactions are an important mediator of therapeutic hASC trafficking.

This work is particularly illustrative of the power and benefit of agent-based modeling in biology. When one examines or studies a biological phenomenon, it is necessary to determine the essential parameters, species, molecules, and behaviors that are necessary or sufficient to account for that phenomenon. Agent-based models can help to formalize this process, and ABMs, like all models (including conceptual models), are necessarily incomplete. Nonetheless, their use helps facilitate rapid discovery; model-aided hypothesis generation provides a systematic means of determining the next steps in the discovery process by identifying what constitutes “sufficient”.

## Results

### Model Verification: ABM Reproduces Key Aspects of Monocyte Trafficking and Ischemia

Two scales of biological organization were present within the model: (1) the cellular-scale encoded at the level of literature-based agent rules (Text S1); and (2) the tissue-level scale represented by the overall model, which was not explicitly programmed and whose observables represent global measurements. Model verification was performed by examining higher order system behaviors (tissue-level scale) and comparing them to corresponding tissue-level measurements from independent wet-lab experimentation. This is, in effect, a test of the effectiveness of the translation of knowledge between the two scales that existed in the model [29].

Specifically, the model was verified by examining both monocyte trafficking ability and key aspects of acute skeletal muscle ischemia, prior to conducting simulations of hASC trafficking. The model reproduced three properties of ischemic injuries including: (1) increases in WSS and network flow rates; (2) up-regulation of key CAM expressed by injured endothelium; and (3) increased secretion of chemokines and cytokines. Similarly, two properties of monocytes were reproduced *in silico* including their independence from selectin-mediated rolling and their average rolling distances during trafficking. These higher order system behaviors were the aggregate result of thousands of interactions between individual agents, their neighbors, and their local environment as they responded to separate dynamic cues outlined in the literature-based rule-sets (cellular-scale).

Ischemic injury was simulated by increasing the pressure at the feeding arteriole by 25%, after which resultant hemodynamic properties were re-calculated (Table 1). Network flow rates were elevated 17%, and average WSS was elevated in arterioles (187%), capillaries (177%), and venules (48%). These hemodynamic changes are characteristic of a collateral microvascular network (adjacent to an injured network) during acute skeletal muscle ischemia. This portion of the network represents one that remains patent following injury and would be capable of supporting the trafficking and extravasation of circulating therapeutic cells (i.e., we are not simulating the vessels downstream of an obstruction that would receive decreased or no blood flow).

**Table 1:** Ischemic injury in a downstream collateral microvascular network was simulated by increasing the pressure at the feeder arteriole by 25%.

*In Silico* Network Parameter	Healthy	Ischemic	Change
**Pressure (mmHg)**			
1 inlet	20	25	25%
5 outlets (mean, min, max)	(16.8, 15, 18)	(16.8, 15, 18)	na
**Network flow (ml/min)**	31.3±69	36.6±70	17%
**Shear stress (dynes/cm^2^)**			
Arteriole (avg)	1.35±1.2	3.89±3.6	187%
Capillary (avg)	0.91±0.5	2.51±1.4	177%
Venule (avg)	1.42±1.3	2.11±2.0	48%
**Location**	Skeletal muscle	Collateral network	na
**Area (mm^2^)**	11.02	11.02	na
**Dimensions (mm)**	4.69×2.34	4.69×2.34	na
**Vessel length per mm^2^**			
Arteriole	0.91	0.91	na
Capillary	2.78	2.78	na
Venule	3.26	3.26	na
**Vessel diameter (µm)**			
Arteriole	10.8±5.05	10.8±5.05	na
Capillary	6.5±1.83	6.5±1.83	na
Venule	12.9±7.81	12.9±7.81	na

The resultant changes in hemodynamic parameters are characteristic of acute ischemic injury in skeletal muscle.

**Figure 2:**
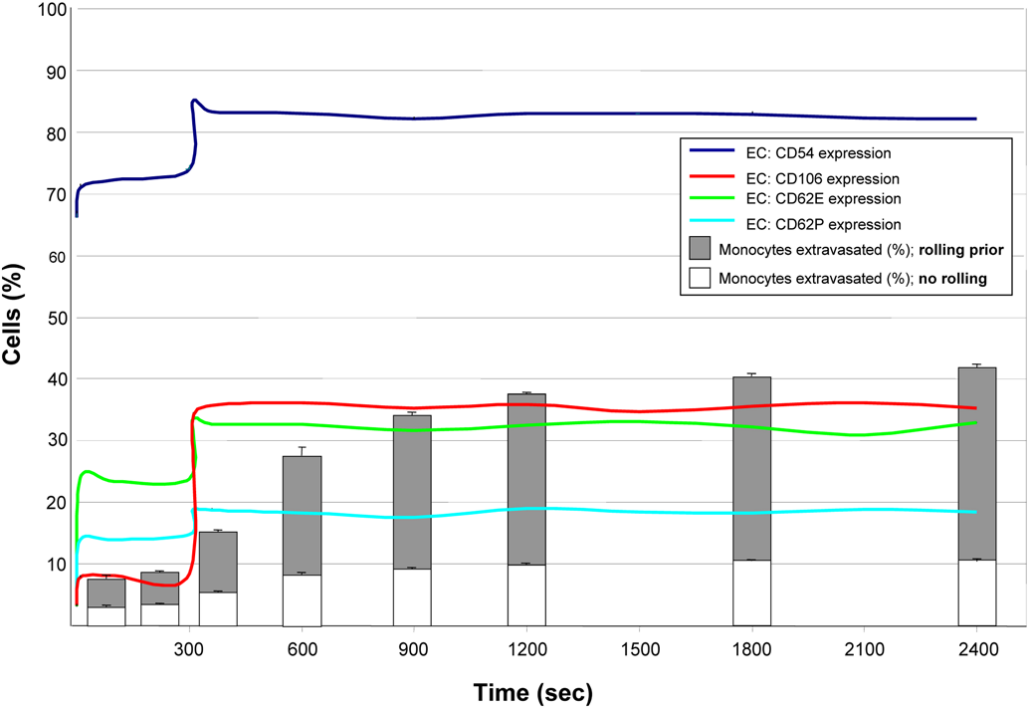
The ABM reproduced the rapid up-regulation of CAM and increased extravasation of monocytes that is characteristic of ischemic injury. Prior to injury at time = 300, healthy endothelium maintained stable CAM expression and low monocyte extravasation. Injured endothelium increased expression of CD54 (ICAM-1), CD106 (VCAM-1), E-selectin (CD62e), and P-selectin (CD62p) following induction of injury (simulated by increasing pressure at sole feeder arteriole by 25%). Monocyte extravasation gradually increased (from 9% to 42%), as did the proportion of extravasated monocytes that rolled prior. All metrics stabilized by 2,400 seconds (40 minutes; n = 5–9). Reported are mean extravasated monocytes+standard deviation. Colored lines depict endothelium CAM expression (average; lines fitted to discrete data points obtained on 10 second intervals).

During acute skeletal muscle ischemia *in vivo*, P-selectin, E-selectin, VCAM-1, and ICAM-1 are upregulated on the luminal vessel wall of the endothelium. In our ABM, all of these CAMs were upregulated by EC agents after the simulated ischemic injury (injury induced at time = 300 seconds). Extravasation of simulated monocytes gradually increased throughout the simulations as increased CAM expression facilitated increased extravasation (Figure 2). Adhesion molecule expression prior to the simulation of the ischemic injury (time = 0–300 seconds) was relatively stable, as expected, and remained stable indefinitely in the absence of an ischemic event (data not shown). Specifically, simulations in “healthy microvascular networks” were performed for 2,400 seconds with and without simulated hASC populations.

**Figure 3:**
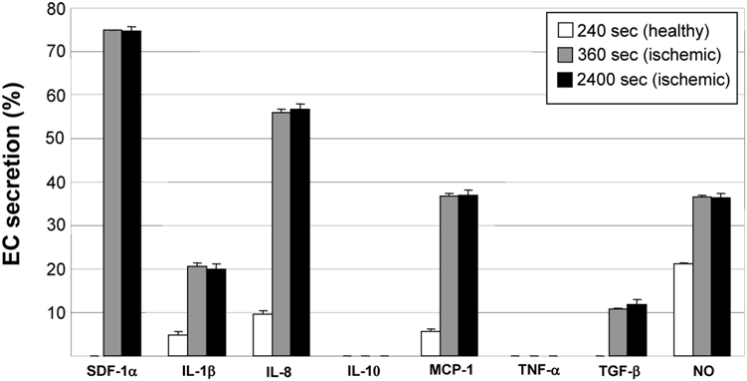
The ABM reproduced increased secretion of inflammatory chemokines and cytokines by simulated endothelial cells. Baseline secretion was relatively low and stable prior to injury at time = 300. Following injury (time = 360), secretion markedly increased and remained relatively unchanged for duration. Changes in secretion of chemokines and cytokines are the aggregate result of cell interactions with each other and their environment. SDF-1α secretion was pre-programmed and not a result of simulated injury, and no rules existed for the secretion of IL-10 or TNF-α by endothelial cells. Reported are mean values+standard deviation (n = 5).

**Figure 4:**
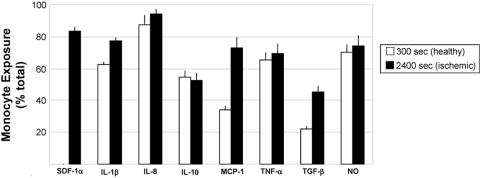
The ABM reproduced increased exposure of circulating monocytes to inflammatory chemokines and cytokines. Prior to ischemic insult, circulating monocyte exposure to soluble and surface-bound chemokines was relatively low (t = 300 seconds; chemokines secreted by monocytes, ECs, tissue macrophages at baseline and WSS-induced changes). After ischemic insult and for duration of simulations (t = 300–2400 seconds), a higher percentage of monocytes encountered chemokines and cytokines, a hallmark of ischemic injury in humans. This led to additional surveying, rolling, firm adhesion, and extravasation by circulating cells. Reported are mean values+standard deviation (n = 5).

Acute ischemic injuries are characterized by alterations in circulating chemokine and cytokine levels in animal models and in humans. Implicated chemokines and cytokines during vascular injury include stromal cell-derived factor-1alpha (SDF-1α), interleukin-1beta (IL-1β), interleukin-8 (IL-8), interleukin-10 (IL-10), monocyte chemoattractant protein-1 (MCP-1), tumor necrosis factor-alpha (TNF-α), transforming growth factor-beta (TGF-β), and nitric oxide (NO), which formed the basis for their inclusion in the rule-sets [4],[5],[30] (Text S1). In ABM simulations, the secretion of inflammatory chemokines and cytokines by ECs was increased (Figure 3). Furthermore, circulating monocytes were increasingly exposed to circulating chemokines and cytokines (Figure 4), as expected. Unfortunately, *in vivo* data for the absolute amount of inflammatory chemokines and cytokines being secreted by ECs during ischemia is inconsistently reported in the literature, and so it could not serve as comparison here. Rather, their increased (relative) presence serves as verification (Text S1).

**Figure 5:**
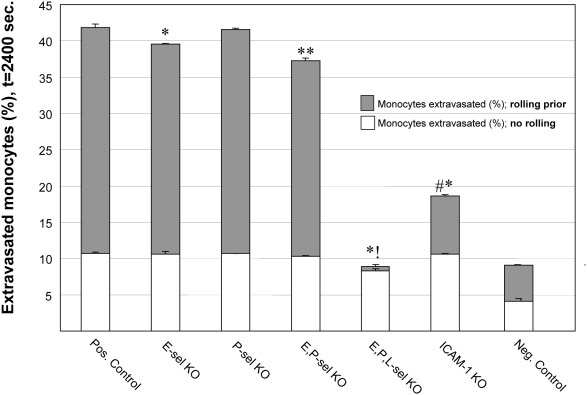
The ABM reproduced expected monocyte trafficking behavior in cellular adhesion molecule knockout experiments. Specifically, levels of monocyte extravasation were unaffected by single selectin knockouts and only marginally affected in simulations of double-selectin knockouts, despite decreases in rolling. Only triple-selectin knockout experiments showed a greater than 80% decrease in monocyte extravasation, which was largely due to an inability to support rolling. Similarly, ICAM-1 knockouts significantly decreased instances of monocyte extravasation, as expected and in agreement with independent experimental findings. Reported are mean values+standard deviation (n = 5–9). Significance asserted by comparing total monocytes extravasated (rolling and non-rolling prior) to the positive control (no knockouts) (p<0.05). Negative controls are included for illustrative purposes only and depict monocytes extravasation in non-ischemic healthy tissue.

**Figure 6:**
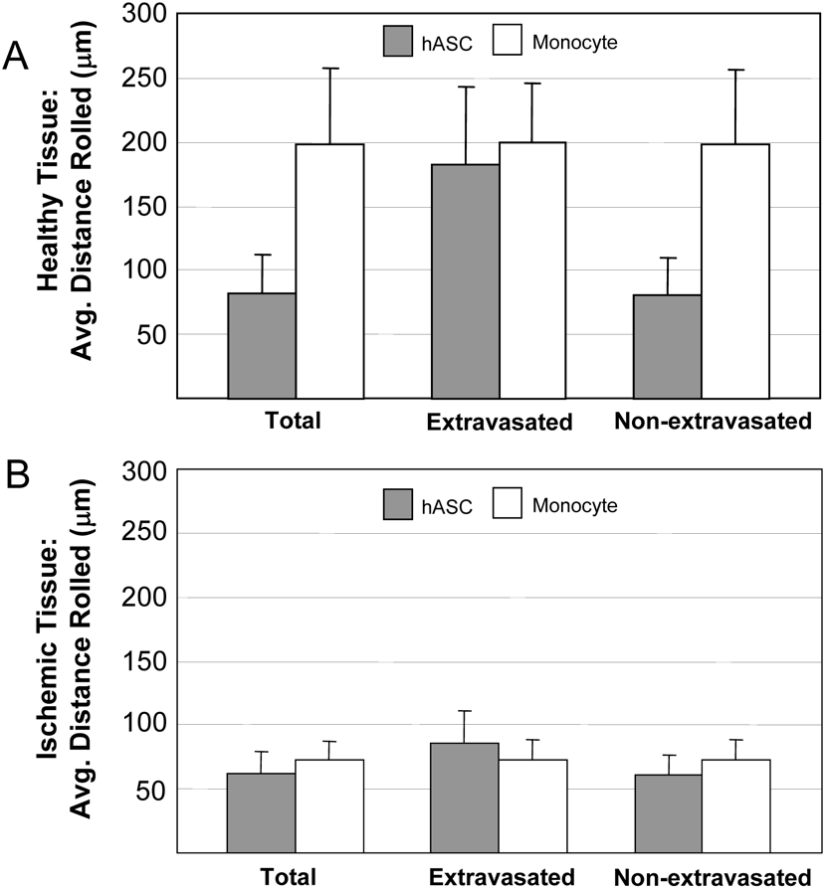
The ABM reproduced appropriate monocyte and hASC rolling distances in simulations of trafficking in both healthy and ischemic tissues. Data presented as the average across all the cells in the phenotype, as well as according to whether cells eventually extravasated. (A) In simulations of healthy microvascular networks, monocytes consistently rolled 200 µm on average. Despite similar rules governing their behavior, hASCs rolling distances varied depending on whether that cell eventually extravasated. (B) In simulations of ischemic microvascular networks, both monocytes and hASC rolling distances were less than levels seen in healthy networks.

Consistent with an earlier ABM [23], monocyte extravasation was shown to be unaffected in E-selectin knockouts, P-selectin knockouts, and only marginally in double selectin knockouts (10% decrease). Triple-selectin knockouts virtually eliminated instances of rolling and extravasation (78% reduction; Figure 5). This agrees with experimental literature showing that monocytes are able to proceed through the adhesion cascade non-sequentially and incorporate into extravascular spaces without rolling on the selectins prior to firm adhesion (Table 2). Simulating a knockout of ICAM-1 significantly inhibited monocyte extravasation (55% reduction; Table 2), and it appears that rolling behavior was affected, as well (Figure 5). Average monocyte rolling distances ranged from 72.6±14.7 to 198±57.5 µm in injured and healthy tissue, respectively. The rolling distances of monocytes that eventually extravasated did not significantly differ from that of monocytes that failed to extravasate, in either healthy or ischemic tissue (Figure 6). *In vivo,* leukocyte rolling distances have been reported to range from 30–400 µm (Table 2).

**Table 2:** Verification of monocyte trafficking behavior.

Verification of Monocyte Trafficking Behavior			
Metric	*In Silico*	Independently Reported Literature Value	Citation
**Extravasation (% reduction)**			
ICAM-1 K.O.	55%	22–30%	[50]–[52]
E-Selectin K.O.	0.5%	30%	[50]
P-Selectin K.O.	0%	0%	[53]
E & P-selectin K.O.	10%	0%	[54],[55]
Triple selectin K.O.	78%	100%	[55]
**Rolling Distance (µm)**			
Healthy Tissue	198.5±57.5	NA	NA
Injured Tissue	72.6±14.7	400; 270±58; 30–80	[52],[56],[57]

Simulations of adhesion molecule knockouts produced reductions in monocyte extravasation, which were in agreement with independent studies. The average rolling distances of monocytes were comparable to values reported in the literature for rolling monocytes *in vivo*. In some cases, independently reported literature values include data derived from other leukocyte subpopulations, *in vitro* data, inhibition studies (not knockout), and/or skeletal muscle exercise model. A 0% reduction may also denote non-significant changes in extravasation or firm adhesion, and/or significant increases in extravasation.

### Model Prediction: hASCs Require Unknown Selectin-Binding Molecule for Extravasation *In Silico*

**Figure 7:**
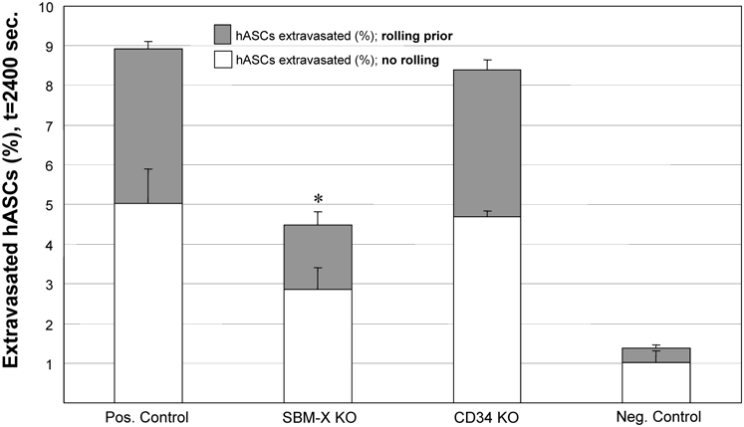
The ABM generated the new hypothesis that selectin-mediated rolling via an unknown adhesion molecule was critical to hASC trafficking. Knockouts of a theorized adhesion molecule, termed SBM-X, significantly reduced hASC extravasation versus positive control (no knockouts). A high degree of hASCs rolled prior to extravasation into ischemic tissue, as compared to the negative controls (non-ischemic healthy tissue). Of note, the elimination of SBM-X from rule-sets also reduced the number of extravasated hASCs that did not roll prior to firm adhesion. Reported are mean values+standard deviation (n = 5–9). Significance asserted at p<0.05.

Within the rule-set, adhesion molecule expression for CD15s, CD34, CD65, E-selectin (CD62e), P-selectin (CD62p), L-selectin (CD62L) and PSGL-1 (CD162) were included for hASC populations (Table 3 and Text S1) because these molecules have all been shown or proposed to support selectin-mediated rolling. Based on independent *in vivo* experiments wherein hASCs were delivered intravenously to ischemic mouse hindlimbs and a review of relevant stem cell literature (Text S1), we expected the efficiency of hASC incorporation into ischemic tissue to be approximately 10% of the total cells delivered, which would also be 3–5× that of levels quantified in non-ischemic tissue. Simulations of hASC trafficking to non-ischemic tissue produced an incorporation efficiency of 1.38±0.43% (i.e., approximately 1–2% of the total injected circulating cells extravasated into the injured extravascular space). Therefore, we anticipated simulations of hASC trafficking to ischemic tissue to produce an incorporation efficiency of 3–10% *in silico*. The rule-sets, as instituted in prototype models, however failed to reproduce this *in silico*; incorporation efficiency was less than 5% (4.49±0.88; Figure 7) and thus at the lower-end of expected values.

**Table 3:** *In Silico* receptor-ligand pairs and functions.

Adhesion Molecule	Ligand	Main Function
LFA-1 (CD11a)	CD54	Firm adhesion & extravasation
MAC-1 (CD11b/CD18)	CD54	Firm adhesion & extravasation
CD15s	Selectins	Capture & rolling
CD29/CD49d (VLA-4)	CD106	Firm adhesion & extravasation
CD34	CD62L	Capture, rolling, & clumping
CD54 (ICAM-1)	MAC-1	Firm adhesion & extravasation
CD62E (E-selectin)	CD15s, CD65, CD162, SBM-X	Capture & rolling
CD62P (P-selectin)	CD162, SBM-X	Capture & rolling
CD62L (L-selectin)	CD34, CD162, SBM-X	Capture, rolling, & clumping
CD65	CD62E	Capture & rolling
CD106 (VCAM-1)	VLA-4	Firm adhesion & extravasation
CD162 (PSGL-1)	Selectins	Capture & rolling
SBM-X (theorized)	Selectins	Capture & rolling

Expanded rules governing selectins, including their expression, ligands, and capabilities were included.

This inconsistency indicated that there likely existed an additional adhesion molecule *in vivo* that was not accounted for by the rules in our initial ABM. We hypothesized that an unknown selectin-binding molecule, similar to PSGL-1, could account for this disparity, and conducted simulations to test this hypothesis by including additional rules in the ABM rule-set. Rules for the hypothesized CAM, termed “selectin binding molecule-X” or SBM-X, included the ability to bind all of the selectins and a 75% probability of expression by hASCs (Text S1). This increased the simulated levels of hASC extravasation to 8.9±1.47% of those delivered (Figure 7), which was in the `expected range. Conversely, knocking out CD34 expression on circulating hASCs *in silico* had no significant effect on extravasation in ischemic tissue (Figure 7), nor did knocking out SBM-X expression on circulating hASCs have a statistically significant effect in healthy tissue (data not shown). Our ABM simulations, therefore, predicted the necessity and ability of therapeutically delivered hASCs to roll on the selectins, independent of PSGL-1.

### Model Validation: hASCs Slowly Roll on Immobilized P-Selectin *In Vitro*

**Figure 8:**
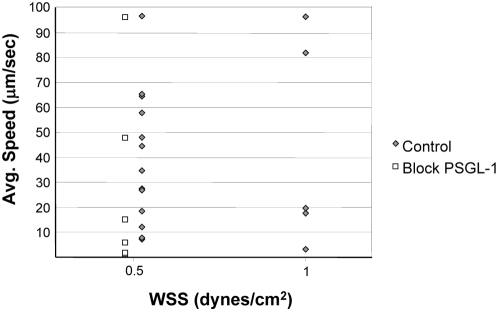
hASCs slowly rolled on immobilized P-selectin *in vitro* at two levels of wall shear stress in the laminar flow assay, instituted in a parallel plate flow chamber. Reported are average quantified speeds of rolling cells below 100 µm/s. Adhesive interactions between hASCs and the substrate both above and below this speed were numerous (data not shown). Each point represents a single observed cell's speed (gray diamonds; data pooled across 4–6 experiments). Interactions, however, were extremely rare and we estimate that less than 1% of hASCs slowly rolled on P-selectin in the laminar flow assay. Competitive inhibition of PSGL-1 was ineffective, as expected (white squares), while blocking immobilized P-selectin with competitive antibodies inhibited all rolling activity (data not shown). Video of the adhesive interactions between hASCs and immobilized P-selectin is available for download at the Peirce-Cottler laboratory website (http://www.bme.virginia.edu/peirce).

**Figure 9:**
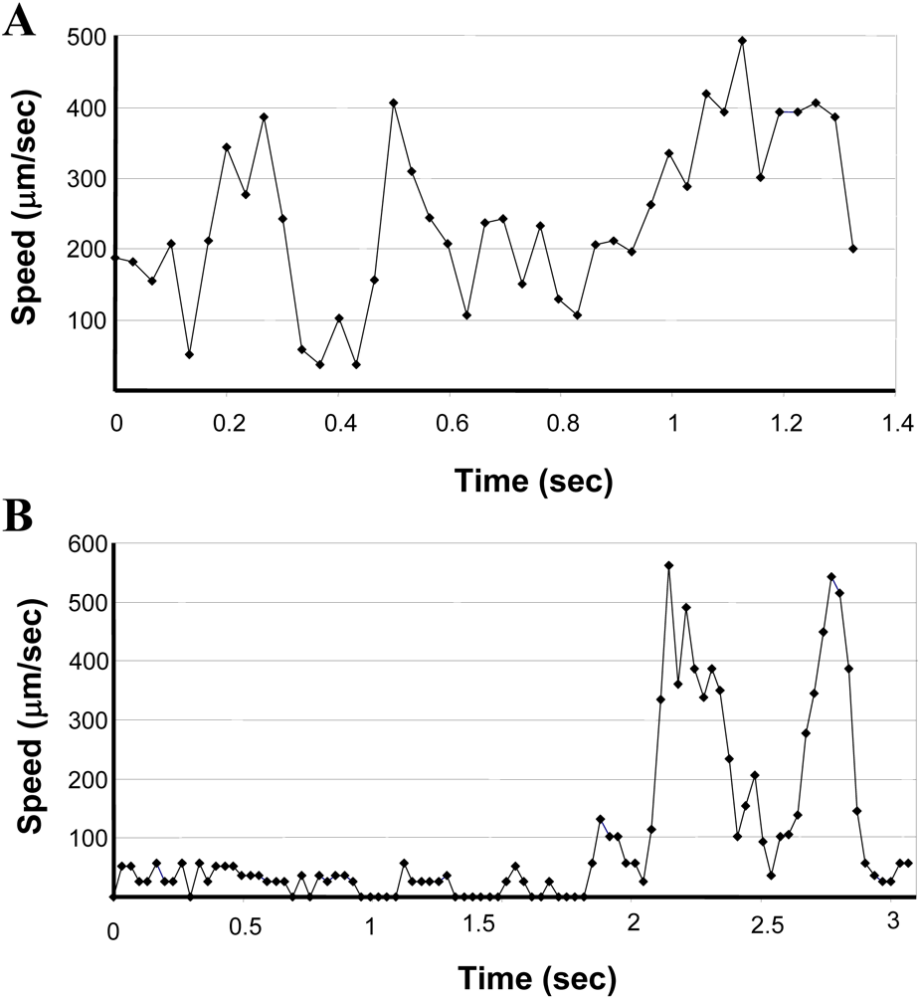
Instantaneous speeds of hASCs that slowly rolled on P-selectin. (A) A fast-rolling hASC exhibited characteristic stop-and-go behavior. (B) A slow-rolling hASC exhibited characteristic stop-and-go behavior. Both representative profiles are of hASCs rolling on P-selectin at a wall shear stress of 0.5 dynes/cm^2^. Non-rolling cells maintained relatively constant speeds (data not shown). Video is available for download on the Peirce-Cottler laboratory website (http://www.bme.virginia.edu/peirce).

The ABM simulations predicted the importance of selectin-mediated rolling for hASC trafficking (Figure 7), despite the fact that hASCs do not express the main ligand for the selectins, PSGL-1. This hypothesis was tested *in vitro* using a parallel plate flow chamber. In the laminar flow assay, where hASCs were perfused over substrates containing immobilized P-selectin, a small percentage of hASCs (<1%) were observed interacting with and slowly rolling on immobilized P-selectin. Rolling speeds as low as 2 µm/s were quantified at two levels of WSS (0.5 dyne/cm^2^ and 1.0 dyne/cm^2^) (Figure 8) and were below that measured for the negative controls (Tween 20 for non-specific adhesion; Fc IgG for human IgG control) and below 20% of the free-stream velocity. The incubation of hASCs with competitive antibodies to PSGL-1 had no effect on hASC rolling speeds (white squares). However, incubating substrates with competitive antibodies to P-selectin eliminated instances of rolling (data not shown), thus confirming that rolling in this assay was mediated by the immobilized P-selectin. Results were consistent across multiple donors and passages (Figure 8). Plots of the instantaneous speeds of rolling hASCs (Figure 9) were typical of rolling leukocytes [31], specifically the characteristic stop-and-go behavior.

### Molecular Mechanism: Surface Expression of CD24 by hASCs

Flow cytometry analysis showed positive surface expression of CD24 by early, middle, and late-passage hASCs (Table 4). Expression was variable and may have been a function of donor and/or passage number. Pooling all data, mean expression level was 6.65±6.7% positive (min = 0.77%, max = 21.1%). Selectin-mediated rolling via CD24 has been shown to occur in other cell populations. This remains to be determined for an hASC population, although constitutive CD24 gene expression has been previously reported [32].

**Table 4:** CD24 expression on hASCs is variable and may be a function of donor and/or passage number.

CD24 Positive hASCs (%)				
	Donor A	Donor B	Donor C	Donor D
P = 1	–	–	13.5	–
P = 2	–	–	–	0.77
P = 3	–	6.8	4.21	1.06
P = 5	21.1	2.41	–	2.48
P = 6	–	7.56	–	–

Pooling all data, mean expression level was 6.65±6.7% positive (min = 0.77%, max = 21.1%). Of note, it was estimated that less than 1% of hASCs were observed to slowly roll.

## Discussion

A better understanding of the homing processes used by therapeutic cells will lead to the development of new and more effective cell-based therapies. For example, the identification of a bottleneck in the adhesion cascade could inform molecular targeting strategies to increase trafficking efficiencies of hASCs following their i.v. delivery during treatment of ischemic injury. And with this study, we now know that approximately 1% of hASCs are capable of slowly rolling on P-selectin, which may limit their homing in pre-clinical studies. Furthermore, this 1% may account for the majority of the cells that are able to successfully home *in vivo*, in which case methods to enhance these P-selectin adhesive interactions should be developed.

These findings need to be verified *in vivo* and their mechanisms of action more clearly elucidated before new therapies can be developed. The development of this and other strategies, however, has been hampered by the complexity of circulating cell homing and the field's incomplete understanding of governing mechanisms. It is true that detailed mechanistic data on the leukocyte adhesion cascade is available, but it is still unclear if the same governing principles universally apply to circulating hASCs. This combined with the fact that investigation of all relevant parameters individually *in vitro* was not immediately feasible (time and resource-prohibitive), prompted us to undertake a computational approach in our study of hASC homing.

We developed an ABM of acute skeletal muscle ischemia and simulated the therapeutic delivery of hASCs. For model verification, we performed a series of *in silico* knockout experiments. Data indicated a prominent role for ICAM-1 in adhesion and extravasation of monocytes, and a less important role for the selectins, which was consistent with independent experimental findings. Furthermore, the model reasonably reproduced key aspects of skeletal muscle ischemia and monocyte behavior, including expected changes in hemodynamics, upregulation of key adhesion molecules on the endothelium, enhanced secretion of inflammatory chemokines and cytokines, and monocyte rolling distances.

Successful ABM verification prompted a series of systematic knockouts *in silico* aimed at identifying bottlenecks in the hASC-specific adhesion cascade. Simulations predicated the necessary expression of an unknown selectin-binding molecule (SBM-X) to achieve expected levels of hASC homing (in contrast to circulating monocytes which were found in the simulation to be insensitive to selectin expression). This does not unequivocally confirm that there were not other mechanisms that would have given this same effect *in silico*, had we hypothesized them (hence the danger inherent in this type of modeling). Rather, the model suggested that hASCs possess the capability to roll on selectins using an un-identified adhesion molecule, in addition to any rolling behavior mediated by CD15s, CD34, CD62p, CD65, E-selectin, or P-selectin expressed on hASCs. Essentially, the failure of the model prompted the formation of a novel hypothesis that we were able to test *in silico* and *in vitro*, which illustrates the power and flexibility of utilizing computational techniques.

The ABM presented here does not accurately reproduce all aspects of acute skeletal muscle ischemia, nor does the rule-set adequately account for the entirety of potential mediators of circulating cell trafficking (e.g., hypoxia, Interleukin-4, proliferation, vasodilation, angiogenesis). It was impractical to account for all aspects *in silico*, and this was not the goal of this first generation ABM. The goal was to develop a computational tool capable of investigating certain aspects of circulating cell trafficking in response to ischemia. This tool was used to generate a new hypothesis, which, when tested in the ABM, informed future experiments. The ABM, therefore, was valuable in that it was able to direct future research by synthesizing available literature, thus instantiating our, and others', hypotheses.

An important part of computational research is the pairing of *in silico* data with independent *in vitro* and/or *in vivo* data [33],[34]. As such, we validated the predictions of the ABM with independent *in vitro* data documenting an ability of hASCs to slowly roll on P-selectin using an adhesion molecule other than PSGL-1. Approximately 1% of perfused cells slowly rolled at speeds as low as 2 µm/s, although it was unclear which adhesion molecule expressed by hASCs was mediating this interaction. Flow cytometry data suggested that CD24, a molecule capable of supporting the rolling of monocytes, neutrophils, and metastatic cancer cells [35],[36], was a likely candidate. Whether CD24 expression by hASCs can support rolling on P-selectin should be verified in future inhibition studies using, for example, a parallel plate flow chamber assay. Additionally, future generations of this ABM should account for CD24 expression levels to determine if this could replace the rule for SBM-X.

Regardless, the ABM was successful for two reasons: (1) simulations informed experimentation that may not have been preformed otherwise; and (2) *in silico* data led to a fundamentally new understanding of hASC biology. For example, data on adhesion molecule expression indicated hASCs do not express PSGL-1, the main ligand for the selectins, at the protein or gene level. It was because of this that the ability of hASCs to undergo selectin-mediated rolling was not investigated *in vitro* until after the ABM suggested otherwise, even though it was known to be important for the trafficking of other circulating cell phenotypes. Most importantly, however, the ABM aided our discovery of the ability of hASCs to dynamically interact with and slowly roll on P-selectin, an adhesion molecule preferentially expressed at sites of injury. This could be significant to the future of injectable hASC therapies, as it suggests molecular targeting and/or sorting strategies to enhance hASC homing. It may also offer evidence for why hASC homing during therapy is so inefficient (i.e., if only ∼1% of hASCs possess a required ability). Collectively, this work illustrates how an ABM can be utilized to inform biological experiments and produce new biological understanding.

## Materials and Methods

For a review of ABMs, please see recent reviews by Thorne et al. [33],[34]. Provided below is a broad overview of the modeling methods that were used. However, Text S1 details the literature-based rules, model construction, model theory, as well as contains in-depth explanations of how the model was implemented and how the rules were formulated. This should be referenced for additional clarification and appreciation of the model building and problem simplification process.

### Agent-Based Model of Acute Skeletal Muscle Ischemia

The ABM of therapeutic stem cell trafficking during acute skeletal muscle ischemia was created through modification of a previously developed ABM of monocyte trafficking in healthy skeletal muscle microvasculature [23]. The most notable changes were a larger microvascular network (approximately 5× larger to include more venules—the presumed site of extravasation), additional cell types (hASCs and tissue macrophages), expanded rules for CAM expression and chemokine and cytokine activity, simulation of acute ischemic injury, and realistic handling of time within the simulation space. The ABM was instituted in the Netlogo software program [27] (version 3.1) on three workstations. Over 150 rules obtained from independent, peer-reviewed literature were used to govern interactions between cells and their environment (Table 5 and Text S1). The ABM's code is available for download at the Peirce-Cottler laboratory website (http://www.bme.virginia.edu/peirce).

**Table 5:** Summary and scope of computational model.

Behavior/Event	Number of Rules/Notes
Cell types	Four: monocytes, hASCs, ECs, macrophages
Acute ischemic injury	25% pressure increase at feeder arteriole
Location	Adjacent collateral microvascular network
Tissue type	Mouse spinotrapezius (skeletal muscle)
Adhesion molecule expression (baseline)	37 rules in three cell types
Adhesion molecule expression (changes)	20 rules in three cell types
Chemokine & cytokine secretion	44 rules in three cell types
Integrin activation	35 rules in three cell types
Survey behavior for circulating cells	11 rules
Population level cell behavior	7 rules in four cell types

In the ABM, agent behavior is governed by literature-based rules derived from independent experimental literature.

**Figure 10:**
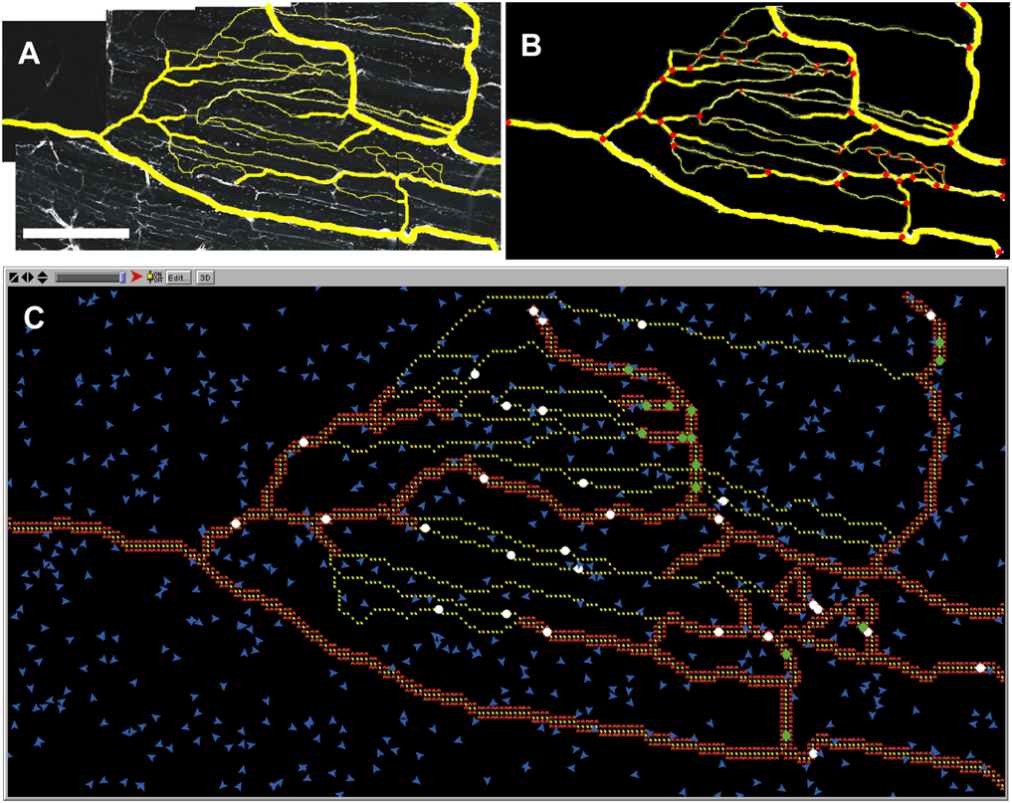
Simulated microvascular network was modeled after mouse skeletal muscle, visualized using confocal microscopy following harvest, using a 20× objective. (A) Confocal microscopy image of mouse spinotrapezius muscle immuno-stained to visualize ECs with BS1-lectin antibody (white). Vascular structures of interest were copied over in yellow in image processing software (ImageJ). Scale bar is 1 mm. (B) The micrograph was manually discretized into nodes, defined as bifurcation points in the microvascular network, and the nodes were connected to form elements. (C) Screen-shot of simulation space. Nodes and elements were manually drawn into the NetLogo simulation space to represent the real microvascular network. Arterioles and venules were characterized on the micrograph based on vessel diameter. Smooth muscle cells are depicted in red lining arterioles and venules. Endothelial cells are depicted in yellow, and tissue macrophages present within the interstitum of the simulation space are depicted in blue.

Skeletal muscle microvascular network architectures for simulations were obtained from mouse spinotrapezius tissues according to previously established protocol [23],[37]. Briefly, mouse spinotrapezius tissues were harvested, immunolabeled, and visualized using confocal microscopy (Nikon, Model TE200-E2 with 20× objective). Endothelial cells were identified by positive isolectin staining (GS-IB_4_ conjugated to Alexa-568; Molecular Probes). Montages of digital images were made, and networks were then discretized into elements (vessels) and nodes (branch-points) before being manually inputted into the simulation space (Figure 10). *In silico* vessels preserved their *in vivo* characteristics, which were stored as agent variables, including vessel phenotype (arteriole, capillary, or venule), vessel diameter, vessel-to-vessel connectivity, and vessel length. Endothelial cell agents were simulated to be 46 µm in length (1 pixel equals 1 endothelial cell), and capillary bed properties were conserved (∼1 mm distance from feeding arteriole to draining venule). The simulation space was discretized into square pixels, and the simulation space was 11.02 mm^2^ (4.69×2.34 mm) containing 1654 endothelial cells (Table 1).

We approached the paradigm of circulating cell trafficking and EC adhesion as being critically dependent on four general components: receptor-ligand interactions, CAM expression, soluble and surface-bound chemokines and cytokines, and hemodynamics. We believed these simplifications were adequate for investigations and development and institution of rules governing these properties are outlined below. For additional clarification and detailed explanation of individual rules, please see Text S1.

### *In Silico* Receptor-Ligand Pairs

**Figure 11:**
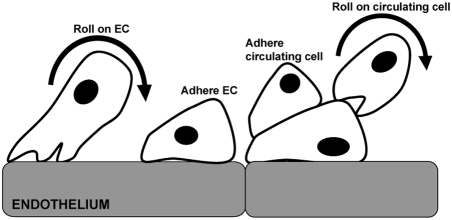
In the ABM of hASC trafficking during ischemia, circulating cells were able to undergo complex adhesive interactions under flow. These included secondary capture: clumping, firmly adhering other circulating cells, and rolling on circulating cells. Rolling along and firm adhesion to the endothelium is present in this model, as well. All of these interactions are of potential importance.

Types of interactions between circulating cells and the endothelium *in silico* included secondary capture and rolling on already adherent circulating cells (Figure 11). Table 3 lists adhesion molecules and binding pairs present within the model. Of note, there were expanded rules governing selectin binding, as this was the primary scope of interest within the ABM. For example, rules governing CD15s [38], CD34 [39], and CD65 [40] expression were instituted in addition to PSGL-1 (CD162) expression because these have been shown to facilitate selectin-mediated rolling. PSGL-1, *in silico* and *in vivo*, is constitutively expressed on monocytes and absent on hASCs. This molecule, or more importantly, the ability to roll via selectins, has been identified as critical for circulating cell homing. Because hASCs do not express PSGL-1, we hypothesized that an additional adhesion molecule (currently unknown) must be capable of supporting slowly rolling on selectins, in addition to any levels supported by CD15s, CD34, E-selectin, P-selectin, and CD65. This hypothesized adhesion molecule was termed “SBM-X” and we assigned identical rules to those that governed PSGL-1 behavior.

### *In Silico* Cellular Adhesion Molecule Expression

CAM expression rules (Text S1) were formulated as the percent probability that an individual agent (cell) within the simulated population would be “positive” for, or expressing, a given adhesion molecule. For example, a synthesis of available literature provided a basis for the rule that 27% of therapeutically delivered hASCs expressed CD54. This rule was applied stochastically, through a random number generator, on an individual basis and averaged to a CD54+ subpopulation representing ∼27% of the total population. CAM expression was dynamic and was re-assessed at a minimum of once every time-step (1 time-step equals 1 second).

Often, multiple publications reported on hASC adhesion molecule expression levels, while also fitting the inclusion criteria (Text S1). In these instances, expression data were averaged to obtain the stated rule. In contrast, the rule for hASC expression of the simulated selectin-binding molecule (SBM-X) was theorized with no basis in the literature. Rather, we hypothesized that additional mechanisms to facilitate selectin-mediated rolling may be required for hASC trafficking, and set SBM-X expression arbitrarily at 75%.

### *In Silico* Chemokine and Cytokine Activity

IL-1β, IL-8, IL-10, NO, SDF-1α, TNF-α, active TGF-β, and MCP-1 were present within the simulation space. These chemokines and cytokines have been implicated in ischemic injuries and/or inflammation 4,5,30 (Text S1).

**Figure 12:**
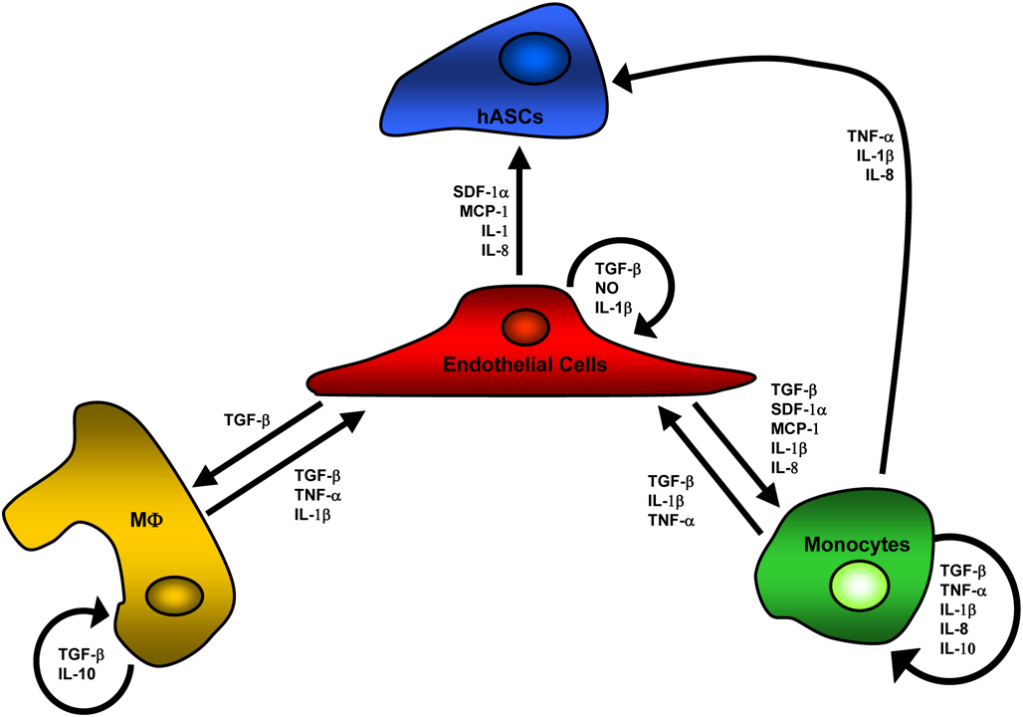
Complex and dynamic connections between agents exist in the ABM. Arrows indicate connections between populations of cells and denote some combination of the following: induced secretion, changes in cellular adhesion molecule expression, and/or integrin activation. These chemokines and cytokines have all been implicated in human ischemic injury, and all connections between cell types are based on relevant, independent, experimental literature.

Chemokine and cytokine activity was simplified to consider only connections between exposure and subsequent behaviors (change in CAM expression, chemokines or cytokine secretion, and/or integrin activation), in a binary manner for each chemokine or cytokine per cell per time-step. This was because: (1) we were concerned with tissue-level changes across large spatial and temporal scales; and (2) data to account for detailed mechanisms (time of secretion, time of exposure, potency, etc.) was not available for all of the chemokines or cytokines. From examined literature, chemokine and cytokine activity reported *in vivo* (both secretion and induced changes in cellular behavior) occurred as a function of location, concentration, time of secretion, time of exposure, diffusion limitations, cell history, cell phenotype, and others. All of this information was not available in the literature for each chemokine or cytokine, making an *in silico* representation of chemokine or cytokine activity this detailed impossible. However generic “promote” or “inhibit” behavior could be deduced from the literature. Therefore, chemokine and cytokine activity *in silico* was simplified in this manner (Text S1) and the emphasis was placed on cause-and-effect linkages between chemokine and cytokine activity and cell behavior (Figure 12). There is precedence for the simplification of cellular behavior in this manner for use in computational modeling [41].

If an agent (cell) were exposed to a chemokine or cytokine *in silico*, it would instantaneously either promote or inhibit a resultant cellular behavior, independent of most of the parameters outlined above, as long as there was evidence in the literature for the connection. What was considered during rule formulation was cell phenotype, history, presence of other chemokines and cytokines, and diffusion limitations.

Consider the following example:

*“Relevant literature was examined. Data reported indicate that if Cell Type A is exposed to Chemokine A, then Cell Type A will secrete Chemokine B. An examination of the methods reveals that only 50% of Cell Type A will respond to Chemokine A at low concentrations, but 65% will respond at high concentrations. Furthermore, secretion of Chemokine B by Cell Type A occurs 30 minutes after exposure to Chemokine A, and ceases 4 hours after removal of the stimulus (Chemokine A exposure).”*

The data from the example would be used to generate the following rule:

“*If Cell Type A is exposed to Chemokine A, Chemokine B is secreted instantaneously (at that time step). Once Chemokine A is removed, secretion is turned off, at that time step.”*

There would also be a percent probability of action assigned to whether Cell Type A would see Chemokine A, and also a percent probability whether Chemokine B would be subsequently secreted.

Whether an agent can become exposed to a secreted chemokine depends, primarily, on its phenotype. For example, circulating cells can become exposed if: (1) they come into contact with an endothelial cell that is actively secreting that chemokine or cytokine (through rolling or firm adhesion); (2) they firmly adhere to or roll over an already adherent circulating cell that is actively secreting that chemokine or cytokine; or (3) they are secreting that chemokine or cytokine (autocrine feedback). In addition to these, endothelial cells can become exposed if a local tissue macrophage present in the interstitium is secreting that chemokine or cytokine.

*In silico*, chemokines and cytokines entered the simulation space through four possible mechanisms: (1) baseline secretion by ECs, macrophages, and/or circulating monocytes; (2) WSS-induced secretion by ECs; (3) chemokine-induced secretion by ECs, macrophages, and/or circulating monocytes; and (4) chemokine or cytokine secretion by ECs local to a site of circulating cell extravasation (trans-endothelial migration). The one exception was secretion of SDF-1α by simulated endothelial cells. At each time step, every endothelial cell in the simulation was assigned a 75% probability of secreting the cytokine SDF-1α, which was then re-calculated at the next time-step. The only evidence in the literature to support this rule was that acute ischemic injuries are often associated with hypoxia, which has been shown to upregulate EC expression of SDF-1α [30]. Future models should account for hypoxia explicitly, and this rule should be flagged for future investigation.

### *In Silico* Hemodynamics

The ABM was paired with a network flow analysis [26], as reported previously [23], in order to calculate relevant hemodynamic parameters. Pressures at the feeder arteriole (inlet) and five draining venules (outlets) were assigned based on a range of physiological values [42], which were then used along with programmed vessel lengths, diameters, and connectivity to calculate pressures, flow rates, and WSS within the microvascular network (Table 1), both before and after simulated ischemic injury. Diameters, vessel lengths, capillary bed size, and pressures were consistent with *in vivo* observations and those found in literature [43]–[46]. For example, in the hASC trafficking model during ischemia, network flow was 31.3 ml/min and WSS in capillaries averaged 0.91±0.5 dynes/cm^2^.

### *In Silico* Survey Behavior

*In silico*, circulating hASCs surveyed their local environments three times every time-step, or every 0.33 seconds. During the “lifetime” of a simulated hASC, each cell surveyed the endothelium a minimum of nine times (assuming complete navigation of vascular network) and at least three times in each vessel phenotype (arteriole, capillary, and venule). It followed that each simulated cell checked its local environment and responded accordingly after each event (e.g., WSS-induced secretion, chemokine-induced secretion, and change in CAM). During rolling and firm adhesion, a simulated circulating cell surveyed many more times; the cell still surveyed every 0.33 seconds, but because it is moving slower than the free-stream, this translated to much more than the minimum of 9 times in its “lifetime”. In theory (but never in practice), every endothelial cell could be surveyed by each circulating cell. This, therefore, represented the maximum, but the exact amount of surveying was dictated by blood flow direction, WSS magnitude, integrin activation, and whether adhesion molecules capable of supporting rolling/adhesion continued to be expressed.

It should be noted that there was little evidence to indicate how often survey actually occurs *in vivo*, or if it does at all. A more realistic representation would be to include absolute receptor numbers, affinity states, and binding kinetics, on a per cell basis. This was not performed in the present generation of the ABM because the focus of this first-generation was on tissue-level changes across entire populations of cells (versus individual cells). During parameterization, however, model outputs were highly sensitive to the frequency of surveying, although relative results remained unchanged (data not shown). To clarify, increasing the survey rate by a factor of ten significantly and substantially increased the degree of both monocyte and hASC extravasation to unrealistic levels, with and without the presence of SBM-X. However, the relative increases in hASC extravasation following the addition of SBM-X in all simulations (low or high survey rate) were consistent.

**Figure 13:**
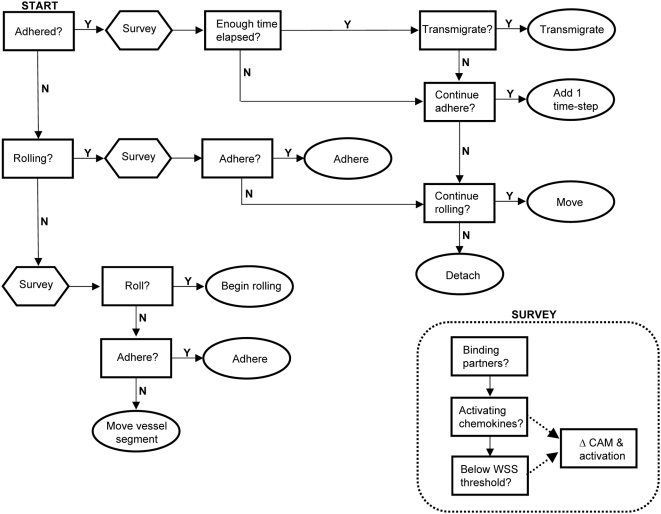
Over-simplified logic diagram of hASC decisions performed at each time-step per agent. For questions (depicted in rectangles) to be satisfied, conditions in rule-set and probabilities of action must be met. These vary according to local conditions. Ovals depict agent actions and signal the end of the time-step. “Survey” block calls the functions listed in the insert. Surveying is preformed at a minimum of each time-step, and if WSS is not below threshold values then the cell will detach or remain in the free-stream (not shown). Not depicted either, exposure to chemokines and/or cytokines is re-set every time-step (all agents), as is chemokine and cytokine secretion (monocyte and EC agents only). Circulating monocytes undergo similar logic, although more complex as they have additional rules concerning chemokine and cytokine secretion, differentiation, and changes in adhesion molecule expression. Endothelial cell agent decisions are dynamic, also. Model code should be referenced for detailed information.

When a circulating agent surveyed its local environment, it “asked” its neighbors whether they were secreting a chemokine or cytokine, what adhesion molecules they were expressing, and what the current WSS was (Figure 13). According to the literature-based rule-sets, if the agent was expressing a complementary ligand, or possessed the ability to respond to an activating cytokine, etc., it would respond accordingly by initiating rolling, firm adhesion, or by doing nothing at all. Once rolling or firm adhesion was initiated, conditions that allowed this to occur must continue at every subsequent time-step for this behavior to continue. For example, a sudden increase in WSS could theoretically detach a firmly adhered agent from the simulated endothelium. Similarly, as a slowly rolling agent moves from one endothelial cell to its neighbor, it must engage appropriate adhesion molecules on the new cell, or it will detach back into the free-stream (Text S1).

### *In Silico* Cell Concentrations

The concentration of simulated hASCs (500,000 cells/0.4 cc PBS) was determined from literature that reported their use as an effective therapy for the treatment of ischemia [20]. This concentration was then diluted in the total blood volume of a mouse, and the rate of hASC generation *in silico* was set to the amount of blood volume passing through the simulated microvascular network, as a function of time and volume flow rate (assuming tail vein injection). Lung and secondary organ entrapment was assumed to be negligible after parameterization identified this as not being a critical parameter (varying entrapment on the first venous pass by 0–25% yielded non-significant differences in the degree of hASC-extravasation).

The rule for tissue macrophage density (∼600 per simulation space) was obtained from literature reporting the number of cells in healthy mouse skeletal muscle microvasculature using immunofluorescence and confocal microscopy [47],[48]. Cell proliferation was assumed to be negligible. Similarly, the number of circulating monocytes was assumed to remain constant during ischemic injury.

### Simulation Execution

The ABM included a realistic representation of time. Each time-step equaled one-second. At every one-second time-step, secretion, adhesion molecule expression, integrin activation, and cell movement (free-stream circulating cells or rolling cells) was assessed, re-calculated, and re-assigned to agents. Each simulation represented 40 minutes (2400 seconds) consisting of five minutes of healthy blood flow followed by 35 minutes of ischemia.

Prior to all simulations, cells were prescribed baseline adhesion molecule expression levels and chemokine secretion profiles, based on the percent probabilities of action that are outlined in the rule-sets (Text S1). The initial tissue location of macrophages was randomly prescribed and re-seeded prior to each simulation, which introduced additional stochasticity. In all instances, flow through a healthy and uninjured microvascular network was simulated for five minutes (computing time approximately 15 minutes). At this time, ischemic injury was applied by increasing the pressure in the feeder arteriole by 25% and hemodynamic parameters (fluid flow, pressure, and WSS) were recalculated within MATLAB. These values were then re-assigned within the *in silico* network, i.v. injection of therapeutic hASCs was simulated, and simulations were continued for an additional 35 minutes (approximately three hours of computing time). In this way, the simulated ischemic injury is one that would be experienced in a collateral microvascular network following acute injury, which is the assumed site of circulating cell extravasation.

Knockout experiments were performed by manually changing the rule-set that each cell (agent) accessed during execution. For example, there was a rule stating that each monocyte will have a 95% probability of expressing LFA-1 (each cell performed this calculation during creation). To interrogate the effects of circulating monocytes that lack this adhesion molecule, this rule would be changed to a 0% probability. Simulations conducted include: (1) E-selectin knockout; (2) P-selectin knockout; (3) L-selectin knockout; (4) E- and P-selectin double knockout; (5) Triple selectin knockout; (6) SBM-X knockout; (7) ICAM-1 knockout; (8) CD34 knockout; (9) Negative control runs where therapeutic hASCs were delivered to healthy skeletal muscle; (10) Positive control runs with all rules in-place.

### Cell Isolation and Culture

The UVa Human Investigation Committee approved all procedures and protocols. Adipose tissue was obtained from either intraoperative suction lipectomy (N = 4) or laboratory liposuction of panniculectomy specimens (N = 4) from 6 female and 2 unknown patients undergoing elective surgical procedures in the Department of Plastic Surgery at the University of Virginia (age range: 20–60; mean age = 40.75 years old). Average body mass index (BMI) was 32.4 (BMI range = 22.5–43.6). hASCs were isolated from harvested adipose tissue using methods previously described [19],[22].

To prevent contamination with hematopoietic cells [49], late passage cells were assayed (p = 3–6) following culture utilizing expansion medium of DMEM/F12 with 10% Fetal Bovine Serum and 1% antibiotic-antimycotic. Plating and expansion medium consisted of DMEM/F12 with 10% Fetal Bovine Serum (FBS) and 1% antibiotic-antimycotic. Cultures were maintained at 37°C with 5% CO_2_ and fed three times per week. Cells were grown to confluence after the initial plating (p = 0), typically within 3–7 days. Once confluent, the adherent cells were released with Trypsin (0.5% Trypsin-EDTA 1×; Gibco; Carlsbad, CA) and then re-plated at 2,000 cells/cm^2^. These cell culture techniques have been shown to prevent contamination with hematopoietic cells by us [19] and others [18], as verified using flow cytometry.

### *In Vitro* Functional Adhesion Assay

The laminar flow assay in parallel plate flow chambers was used to verify ABM predictions, specifically to determine whether hASCs possessed the capability to slowly roll on P-selectin under flow conditions. Experiments were performed according to established protocol [22]. Briefly, P-selectin (Recombinant human CD62P Fc chimera; R & D Systems; Minneapolis, MN) was immobilized to the substrate (polystyrene tissue culture plastic) by diluting the protein in PBS (10 µg/ml), followed by a 3 hour incubation at room temperature, and an overnight incubation at 4°C with 0.5% Tween 20 in PBS. hASCs (suspended in PBS; 0.1 mM Ca^2+^ and Mg^2+^) were then perfused over the substrate (incorporated as the lower wall in the parallel plate flow chamber) at WSS of either 0.5 dynes/cm^2^ or 1.0 dynes/cm^2^, as indicated, and interactions were observed using an inverted phase contrast microscope (Diaphot-TMD; Nikon, Garden City, NY) at 10× magnification. Inhibition studies were performed by either: (1) incubating hASCs with competitive antibodies to PSGL-1 (clone KPL-1; Abcam, Cambridge MA) for 30 minutes prior to assay; or (2) incubating substrates with anti-P-selectin competitive antibodies (10 µg/ml; monoclonal anti-human CD62p; clone G1/G1-4; Ancell, Bayport MN) for three hours at room temperature after immobilizing P-selectin. To rule out non-specific interactions, negative control experiments were performed with recombinant human IgG_1_ Fc (10 µg/ml; R & D systems, Minneapolis, MN) immobilized to the substrate, as well as 0.5% Tween 20 diluted in PBS.

### Data Acquisition and Analysis during Functional Adhesion Assay

A Kodak MotionCorder Analyzer model 1000 camera (Eastman Kodak, Motion Analysis System Division, San Diego, CA) was used at a frame rate of 30 frames/second. A minimum of 30 rolling cells were analyzed to calculate average speeds. Cell interactions analyzed using VirtualDub (Version 1.6.19.0; Copyright 1998–2007 by Avery Lee; http://www.virtualdub.org) and Xoomer (Version 1.3; http://www.xymantix.com) software programs.

### Flow Cytometry

Following trypsinization, hASCs (N = 4 donors) were evaluated for surface expression of the protein CD24 (EbBiosciences; San Diego, CA) using flow cytometry using a Becton Dickinson FACS Calibur (Franklin Lakes, NJ). Cell viability was greater than 98% (Trypan Blue dye; Gibco), and a minimum of 10,000 events were counted for each analysis, including isotype-matched controls (PE Mouse IgG1 K isotype; Ebioscience, San Diego CA) and positive control (HLA-ABC; Abcam, Cambridge MA).

### Statistics

All statistical comparisons were made using the statistical analysis tools provided by SigmaStat 5.0 (Systat, Inc., Point Richmond, CA). Results are presented in the form of mean±standard deviation, and statistical significant was asserted at p<0.05. In terms of the reported sample sizes, “N” refers to the number of individual donor patients from which hASCs were harvested, whereas “n” refers to the number of independent trials (replicates) that were run with a particular assay condition or simulation. Raw data were tested for normality, before being analyzed using 1-way ANOVA followed by Tukey's t-test. Parallel plate flow chamber studies were normalized to negative control runs using Tween 20, with the same cell populations. Data from knockout simulations were assessed compared to positive controls, where all rules for ischemic injury are intact (negative controls are included for reference only; no statistical relevance).

## Supporting Information

Text S1 Detailed explanation of rule formulation and execution, model logic, and literature-based rules.
(0.61 MB DOC)
