# Supplementary material for: Agent-Based Model of Therapeutic Adipose-Derived Stromal Cell Trafficking during Ischemia Predicts Ability To Roll on P-Selectin
Source: PLoS Comput Biol. 2009 Feb 27;5(2):e1000294. doi: 10.1371/journal.pcbi.1000294 (PMC2636895; doi:10.1371/journal.pcbi.1000294)
Supplement: Text S1 — Detailed explanation of rule formulation and execution, model logic, and literature-based rules. (0.61 MB DOC) [file pcbi.1000294.s001.doc]

**Supplemental**

*In silico*, agent behavior was governed by literature-based rule-sets obtained from peer-reviewed independent literature (Tables S1-S4). Derivation of rule-sets in any ABM is inherently subjective, although in their entirety should accurately reflect the current understanding in the field. This section outlines the criteria and methods employed to generate the rule-set, as well as how they were instituted within the Netlogo [1] software program. Also included are tables of rules that governed interactions *in silico* (Tables S1-S4), which should be consulted for explanation of the ABM, and a simplified logic diagram of hASC agent decisions (Figure 13). Of helpful reference may be a prior model [2] that served as the basis for the development of this model (available for download on the Peirce-Cottler laboratory website; [www.bme.virginia.edu/peirce](http://www.bme.virginia.edu/peirce)).

hASC adhesion molecule expression (Table S1)

For experimental data to fit inclusion criteria for rule formulation:

1. Cells must be of human origin obtained either through liposuction or lipectomy procedures and be early to mid-passage (p=0-4) at point of assay.
2. Data obtained by testing fluorescence using flow cytometry.
3. Data obtained from independently published peer-reviewed scientific articles with a single exception: PSGL-1. This rule (0% probability of expression) was confirmed in-house (data unpublished).

Monocyte adhesion molecule expression (Table S1)

Monocyte-endothelium interactions during the adhesion cascade have been intensely studied and reported in the literature. In most instances, there were too many high-quality studies to cite individually for each adhesion molecule expression rule. Here, the reader was referred to a relevant review or text. Regardless of whether data indicated constitutive expression (100% probability of expression), the instituted rule within the simulation space was set at a 95% probability of positive expression, at baseline. This maintained a level of randomness in the system.

For experimental data to fit inclusion criteria for rule formulation:

1. Cells must be monocytes of human origin, isolated from whole blood.
2. Data obtained by testing fluorescence using flow cytometry.
3. Ideally, cells obtained from healthy patients.

There were exceptions. The rule for CD65 expression (25% probability of expression) was derived from data obtained examining peripheral blood monocytes isolated from healthy patients. In this study, only mean fluorescence intensity was reported making it necessary to normalize to CD11a expression (assumed to be constitutively expressed). Similar techniques were utilized for the generation of other adhesion molecule expression rules, when necessary.

Endothelial cellular adhesion molecule expression (Table S1)

For experimental data to fit inclusion criteria for rule formulation:

1. CAM expression determined using fluorescence and/or confocal microscopy.
2. Tissue source in order of preference: human endothelium obtained from healthy skeletal muscle microvasculature, human endothelium obtained from healthy coronary microvasculature, murine skeletal muscle microvasculature, murine coronary microvasculature.
3. CAM expression normalized to PECAM-1 (CD31) or other constitutively expressed endothelial cell marker, when necessary.

For rule formulation, it was not ideal to use data from *in vitro* studies if cell lines were immortalized or of umbilical vein origin because they are phenotypically different from skeletal muscle endothelium. *In vitro* data was also problematic if it was obtained from statically cultured endothelial cells. Judging endothelial cell CAM expression independent of fluid flow forces does not reproduce *in vivo* conditions that have been shown to be critical in CAM regulation/expression.

However, it was necessary to consult *in vitro* data or less-than-ideal *in vivo* data when other data was not available. For example, during rule formulation for PSGL-1 expression, a study was referenced where human umbilical vein endothelial cells (HUVECs), foreskin microvascular ECs, and atherosclerotic arteries were assayed using immunofluorescence. For previously stated reasons, this was a weaker rule and should be flagged for future parameterization or *in vivo* investigation. Similarly, the rule for P-selectin (CD62p) expression was formulated from data obtained from rat heart microvasculature and mouse skeletal muscle microvasculature.

In most instances, endothelial cell adhesion molecule expression was verified in other systems including retinal microvasculature, brain endothelium, or *in vitro* (protein or mRNA expression). These studies add additional validation to the formulation of the rule-set, but were not cited for reasons stated previously.

Tissue macrophage and smooth muscle cell adhesion molecule expression

There were no rules governing CAM expression of either tissue macrophages or smooth muscle cells. In the simulation space, tissue macrophages did not migrate or travel through the circulation; hence these rules were not necessary. Likewise, smooth muscle cells did not migrate or travel through the circulation. Instead, they served only to visually distinguish between vascular phenotypes. Future models should consider inclusion of additional rules to account for these cell types.

Chemokine secretion (Table S2)

Upon creation within the simulation space, each agent was assigned a percent probability of secreting each accounted-for chemokine (0-95% probability of secretion). Rules were accessed by every agent, at every time-step, and if probability was met (random number generator) then secretion was assigned as “positive” (conversely “negative” if probability was not met). If positive, secretion occurred instantaneously at that time-step (must be re-calculated at every time-step; instantaneously off).

For formulation of rules for baseline chemokine secretion, simplifications were necessary. We considered predominately cell phenotype. For example, during ABM construction the question was asked: “Have microvascular endothelial cells been shown to secrete IL-1β  independent of external stimuli?” Answers to these questions were sought for every chemokine and every cell phenotype present within the model, except for hASCs and smooth muscle cells (no programmed chemokine secretion ability).

Basis for answers to these questions as being “positive” or “negative” were found in relevant literature and were cited in the rule-set. Data from literature was then synthesized to assign relative percent probabilities of secretion on a per cell basis. For example, parameters such chemokine concentration, length of secretion, etc. were used to group secretion rules according to a low to high probability of action (10% to 75%). These were not detailed rules; in most instances a general review of chemokine activity during ischemia could be referenced for verification.

Chemokine-induced chemokine secretion (Table S2)

In the simulation space, if an agent was exposed to a chemokine, it might have induced secretion of additional chemokines (assuming there is a rule and basis in the literature for this connection). During rule formulation, connections between exposure to specific chemokines and subsequent chemokine secretion were emphasized. This means that rules stated a chemokines’ ability to promote or inhibit behavior of specific cell types only.

Key criteria for rule formulation:

1. Evidence was found in relevant literature for connections between a specific chemokine and its ability to induce secretion of additional chemokines.
2. The effect of exposure to a specific chemokine was simplified to consider only whether it promoted, inhibited, or had no effect on additional chemokine secretion.
3. The ABM accounted for synergistic effects. For example, certain chemokines only worked in the presence of other chemokines.
4. Only data that quantified changes in protein expression or secretion were considered. *In vivo*, a change in mRNA expression does not necessarily translate to a change in protein secretion.
5. A rule for inhibition reflected experimental data that indicated blocking secretion, blocking a receptor, down-regulating a receptor, etc. Likewise a rule for promotion indicated changing affinities, upregulation of receptors, additional secretion, etc.
6. We developed a computational tool to investigate acute skeletal muscle ischemia. As such, data from literature were only used if effects were seen at less than eight hours, though preferably four hours, after initial stimulus.

Key criteria governing implementation in the simulation space:

1. An agent must “see”, or be exposed, to a stimulus prior to any induced secretion. These were parameterized rules dependent on cell phenotype and location. For example, an endothelial cell would “see” a stimulus if either a) surrounding EC in the same vessel segment was secreting the chemokine, b) a circulating monocyte that was secreting a chemokine has rolled or firmly adhered to that EC, or c) a tissue macrophage in the interstitium local to the vessel was secreting a chemokine. If these conditions were met, then a probability of 25% was assigned to whether that EC would “see” the stimulus. This was calculated for every cell within the simulation, at every time-step.
2. Once an agent had “seen” a stimulus, there was a probability of action associated with that cell’s response (i.e., promote or inhibit further secretion). This was set at 50% for all cell phenotypes (assuming there was a rule governing action).
3. Chemokine secretion occurred instantaneously if conditions were met, and after every time-step, chemokine secretion and chemokine exposure (“see” a stimulus) was turned off instantaneously (binary). In this way, the continued presence of activating stimuli was necessary for secretion to continue.
4. At every-time step, if additional activating stimuli were not “seen” or if secretion was not induced, cells would return to baseline secretion levels.

Rule-set: WSS-induced chemokine secretion (Table S2)

Wall shear stress-induced chemokine secretion was instituted within the simulation space in a manner similar to chemokine-induced chemokine secretion. Rules were simplified to consider only connections between changes in WSS and the resulting secretion. Unfortunately, the majority of available data in the literature was collected from statically cultured cells that experienced sudden changes in WSS. Ideally, data from cells that had experienced long-term culture under flow conditions before experiencing changes in WSS would have been assayed instead. Similarly, there were many studies that examined changes in CAM expression following induction of flow *in vitro*, but again, this was not sufficient in that it fails to distinguish between observed changes due to the onset of flow and those due to changes in the magnitude of flow.

Key criteria for understanding implementation in the simulation space:

1. Only endothelial cell agents undergo WSS-induced chemokine secretion. No other agent phenotype (cell type) was capable of sensing changes in WSS *in silico*.
2. Secretion was instantaneously “on” if correct stimuli were present (probability of action was met), as well as instantaneously “off” if fluid flow forces were removed.
3. The probability of secretion if changes in WSS were sensed was assigned to a relative scale between 10% and 80%, depending on the magnitude of change. This subjective handling considered magnitude of WSS, previous levels of WSS, length of secretion, and amount of chemokine secreted, as reported in the literature (assessed as: low, medium, high, and very high).
4. WSS-induced secretion occurred only during periods of changing WSS (e.g., at the onset of acute ischemic injury). This accounted for the phenomena of de-sensitization. Nitric oxide (NO) secretion was the exception (NO was secreted under baseline conditions in healthy skeletal muscle microvasculature, *in silico*).
5. When EC agents experienced changes in WSS, however, NO secretion changed well, similarly to other chemokines.

Transmigration-induced chemokine secretion (Table S2)

*In silico*, a transmigrating monocyte or hASC induced secretion of chemokines from local EC agents. During each event, the probability of secretion occurring was set between 10% and 40% for each surrounding EC (chemokine-specific). Rules were based on *in vitro* assays where monocytes and ECs were co-cultured together and secretion of chemokines into the media was quantified. Reported are synergistic effects; neither cell was able to secrete similar levels without cell-cell interactions. The probability of action was set on a relative scale, considering cell number, degree of interaction, concentration of secreted chemokine, and time-span of secretion reported in the literature. For these rules, hASCs were assumed to behave as monocytes.

WSS-induced change in adhesion molecule expression (Table S3)

Rules were similar to those that governed WSS-induced chemokine secretion. Again, only changes in WSS were considered relevant. *In silico,* expression of CD54 and CD106 by EC agents were affected, and only occurred at the onset of ischemic injury and times after (coinciding with abrupt change in experienced WSS). It was instituted by changing the baseline expression level of these CAMs to 75% probability of expression for CD54 and 30% probability of expression for CD106. It is these new baseline-expression levels that each EC agent defaulted to, at every time-step, if other additional activating stimuli were not seen. There was discrepancy in the literature on whether an increase in WSS induced changes CD106 expression. A high-confidence rule was difficult to formulate because of reasons stated earlier (statically cultured ECs are inherently different than ECs cultured under flow). Nonetheless, in this model an increase in WSS increased the probability of positive CD106 expression. However, changes in WSS also induced increased secretion of NO, which mitigated this response (NO inhibited CD106 expression *in silico*). Future *in vivo* experimentation should be done to formulate higher confidence rules, and the effects of NO on other CAM should be included in future models.

Integrin activation (Table S4)

Prior to transmigration *in silico*, a circulating cell generally had to proceed through a several events first. A circulating cell would first survey its local environment to assess whether binding ligands were present. Second, the combined influences of chemokine and hemodynamics would force interactions and facilitate further survey behavior. At this point, for firm adhesion and transmigration to proceed, integrin activation must occur (firm adhesion without integrin activation was possible, although rare). Physiologically, integrin activation *in silico* represented the conformational change of integrins present on circulating cells into a high affinity state (LFA-1, MAC-1, and VLA-4). Once this occurred, firm adhesion was more likely to occur. Once firmly adhered, a cell had to remain firmly adhered for 10 + 2 seconds before initiating transmigration (during that time, continued CAM and chemokine presence was necessary to maintain adhesion, at every time step). There was a probability of action assigned to integrin activation and transmigration, derived from independent experiments published in relevant, peer-reviewed literature. Conversely, the rule for the elapsed time necessary for transmigration to occur was scaled relative to the time required for other events, and this relative scaling was based on accepted dogma in the field. In that sense, it was arbitrary and not explicitly based on relevant literature.

Key for understanding rule-creation and *in silico* execution:

1. Integrin activation and transmigration occurred instantaneously.
2. Before a cell could proceed from firm adhesion to transmigration, it had to survey its environment and ensure that appropriate adhesion molecules and chemokines were still present. Most circulating cells remained firmly adhered for ten time steps, which was enough time for local environment conditions to change (hence necessity to re-survey).
3. There was a probability of action associated with integrin activation, firm adhesion, and transmigration as a function of chemokine presence, cell phenotype, cell history, hemodynamic forces, adhesion molecule expression, binding molecules present, and time.
4. A cell that was firmly adhered but failed to transmigrate was either released back into the free-stream (33%), initiated rolling along endothelium (33%), or remained firmly adhered for at least one additional time-step (33%). Continued firm adhesion and rolling was contingent upon appropriate external chemokines and adhesion molecules being present.
5. Integrin activation on hASCs was modeled after integrin activation on monocytes. This simplification was necessary because data was not available.
6. Rules were formulated from data taken from parallel plate flow chamber assays where adhesion molecule expression on substrates, WSS, and chemokine presence was tightly controlled. Data reporting percent of cells that firmly adhered under prescribed conditions was used to formulate *in silico* rules. For example, if *in vitro* experiment reported that when monocytes were flowed over the protein VCAM-1 under specific conditions, 45% of the cell population would firmly adhere, a rule was instituted where, if similar conditions were present, a single monocyte had a 45% probability of adhesion. In this way, each individual cell behaved according to its local environment and individual characteristics.
7. When experimental literature reported an increased ability of a circulating cell to firmly adhere following chemokine activation, this was instituted *in silico* as a promotion of integrin activation. However, data *in vitro* may have been the result of changes in the receptor, ligand, or other undetermined mechanism. This should not influence the outputs of the model, but should be considered before interpreting results. For example, if simulations reported the importance of SDF-1α activation during trafficking, it should be remembered that the ABM does not differentiate between effects on the receptor or effects on the ligand.
8. Occasionally, it was necessary to formulate rules for integrin activation from data on neutrophil activation. However, it is likely that integrin behavior is conserved across leukocyte sub-populations. These rules, however, should be updated as additional experimental data becomes available.

hASC incorporation into ischemic tissue *in silico* (Table S5)

The inability of the model to reproduce hASC incorporation efficiencies of 3-10% into ischemic tissue (and 3-5x that of levels quantified in healthy tissue) led to a re-examination of the model’s underlying literature-based rules and, ultimately, the generation of a new hypothesis. As such, it is important to examine and justify: 1) why these incorporation levels were anticipated within the simulation space (i.e., do these data accurately reflect the current understanding in the literature?); 2) why hASC ability to undergo selectin-mediated rolling was investigated further, instead of other steps in the adhesion cascade; and 3) whether the parameterization and subsequent adjustment of other literature-based rules could have restored appropriate levels of incorporation, as well.

First, a survey of all known relevant literature reporting the use of hASCs in the treatment of ischemic injuries was performed (Table S5). In most instances, incorporation efficiencies were not reported or were not applicable to the simulations. For example, incorporation was not assayed at early time-points (incorporation may decrease as time increases), tissues were not perfused prior to harvesting to eliminate circulating cell contamination (cell counts may be artificially high), or it was difficult to confidently extrapolate tissue-level incorporation from cell counts in relatively few tissue sections (counts may be artificially low). For these reasons, much of the data surveyed was not suitable for comparison to *in silico* simulations. To complement the hASC data, studies reporting the use of other similar cell types were referenced. However, no consensus existed here, other than an agreement that incorporation following therapeutic delivery was variable and low. Bone marrow mononuclear cell and endothelial progenitor cell incorporation in models of hindlimb ischemia varied from 7-20% [7-9], and incorporation in a mouse model of myocardial infarction varied from 2.6-4.7% (dependent on delivery method; [10]). Similarly, incorporation efficiencies into injured tissue following myocardial infarction in human patients varied from 1.3-39% (dependent on cell phenotype and patient characteristics; [11]). These wide data ranges were all considered when formulating model expectations; it was believed that hASC incorporation efficiencies should be significantly greater than that seen in healthy limbs (3-5x greater) and represent approximately 10% of the total delivered cells. This was based on the following:

- 1. The most reliable hASC data was comparing cell counts in the ischemic limb to cell counts in the ischemic limb (Table S5; “specificity”). Tissue was assayed at 48 hours, which we anticipate would trend lower than tissues being assayed at less than 1 hour.
  2. There may be overlapping ranges of incorporation efficiencies between hASCs and other related therapeutic cell types, in a variety of types of ischemic injury.
  3. A “yes/no” binary response, with an order of magnitude difference between negative incorporation and positive incorporation was reasonable.
  4. The simulation space in our ABM included only one microvascular network and not an entire tissue.

The authors recognize that these assumptions may have to be re-visited in future models; it was difficult to compare *in silico* incorporation levels to *in vivo* incorporation levels with absolute certainty (i.e., which independent data-set to verify simulation results with?). However, it is important to note that the inclusion of SBM-X in simulations significantly enhanced incorporation efficiencies over control runs where SBM-X did not exist. Thus, it was the relative increase in incorporation efficiency that prompted the formation of a new hypothesis concerning the rolling ability of hASCs.

Rolling during the adhesion cascade was investigated further because it was known that this was shown to be a bottleneck and rate-limiting step for the homing of neutrophils, and it was thought this could be the case for hASCs as well. Furthermore, it was interesting that hASCs do not express PSGL-1 (critical for leukocyte homing) and still traffic to ischemic tissues and confer a therapeutic benefit following i.v. delivery (Table S5). It was conceivable that inadequacies in the rules that govern firm adhesion and/or chemokine and cytokine secretion could have contributed to the low incorporation efficiencies, but this was believed to be a low likelihood. This was for the following reasons: 1) these rules were formulated with a higher confidence, based on a greater number of relevant published studies; and 2) the rules governing chemokine and cytokine behavior were simplified to consider only connections between cell-types (ignored potency, concentration, timing, etc.) and thus were high-confidence rules, as well. Nonetheless, systematic chemokine knockout experiments were performed *in silico* [12]. The possibility that more that one inadequacy within the rule-set could have theoretically restored expected incorporation efficiencies further emphasizes the importance of pairing all computational simulations with *in vitro* and/or *in vivo* experimentation [13].

**Supplemental Tables**

| **Adhesion molecule** | **Cell** | **Positive Population** | **Citation** |
| --- | --- | --- | --- |
| **LFA-1 (CD11a)** | hASC | 1% | [3,4] |
|  | Monocyte | 95% | established; [5-7] |
|  | EC | 0% | assumed |
| **MAC-1 (CD11b/CD18)** | hASC | 27% | [3,8] |
|  | Monocyte | 95% | established; [5-7] |
|  | EC | 0% | assumed |
| **CD15** | hASC | 3% | [9] |
|  | Monocyte | 90% | [7] |
|  | EC | 0% | assumed |
| **VLA-4 (CD29/CD49d)** | hASC | 55% | [3,10-12] |
|  | Monocyte | 95% | established; [6,7,13] |
|  | EC | 0% | assumed; [14] |
| **CD34** | hASC | 52% | [3,9,12,15] |
|  | Monocyte | 0% | assumed |
|  | EC | 77% | [16,17] |
| **CD54 (ICAM-1)** | hASC | 27% | [3,8] |
|  | Monocyte | 25% | [6,18,19] |
|  | EC | 66% | [16] |
| **CD62E (E-selectin)** | hASC | 2% | [3,20] |
|  | Monocyte | 0% | assumed; [5] |
|  | EC | 6.5% | [16] |
| **CD62L (L-selectin)** | hASC | 0% | [4,20] |
|  | Monocyte | 90% | [21,22] |
|  | EC | 0% | assumed |
| **CD62P (P-selectin)** | hASC | 0% | [4] |
|  | Monocyte | 0% | assumed; [5,23] |
|  | EC | 5% | [16,24,25] |
| **CD65** | hASC | 29% | [9] |
|  | Monocyte | 25% | [26] |
|  | EC | 0% | assumed |
| **CD106 (VCAM-1)** | hASC | 15% | [4,11] |
|  | Monocyte | 0% | assumed; [5] |
|  | EC | 3.5% | [16] |
| **CD162 (PSGL-1)** | hASC | 0% | Verified experimentally |
|  | Monocyte | 95% | [27] |
|  | EC | 10% | [28] |
| **SBM-X** | hASC | 75% | Theorized |

**Table S1.** Baseline CAM expression for monocytes, endothelial cells, and hASCs as implemented within the ABM.

| **Chemokine**  **Secreted** | **Activator** | **Responder** | **Stimulus: Probability** | **Citation** |
| --- | --- | --- | --- | --- |
| **IL-1β** | Baseline | Monocyte / MΦ | 40% | [29-33] |
|  | Chemokine-induced | EC  EC  Monocyte  Monocyte  MΦ | IL-1β: promote  TNF-α: promote  IL-1β: promote  MCP-1: promote  TGF-β: inhibit | [30,34-36] |
|  | Δ in WSS-induced | EC | Increase: promote | [37] |
| **IL-8** | Baseline | EC  Monocyte | 20%  60% | [32,38-40] |
|  | Chemokine-induced | EC  EC  Monocyte  Monocyte | IL-1β: promote  TGF-β: inhibit  IL-1β: promote  TNF-α: promote | [40-42] |
|  | Δ in WSS-induced | EC | Increase: promote | [43,44] |
|  | Transmigration | EC | 40% | [38] |
| **IL-10** | Baseline | Monocyte | 20% | [32,45] |
|  | Chemokine-induced | Monocyte  Monocyte  MΦ | TNF-α: promote  IL-10: inhibit  TGF-β: promote | [45-47] |
| **TNF-α** | Baseline | MΦ  Monocyte | 30%  60% | [30-32] |
|  | Chemokine-induced | Monocyte/MΦ  MΦ  Monocyte | IL-10: inhibit  TGF-β: inhibit  TGF-β: promote | [30,34,48] |
| **MCP-1** | Baseline | EC | 60% | [32,38] |
|  | Chemokine-induced | EC  EC  EC  MΦ | TNF-α: promote  IL-1β: promote  TGF-β: inhibit  TGF-β: promote | [49,50] |
|  | Δ in WSS-induced | EC | Increase: promote | [51] |
|  | Transmigration | EC | 10%  with IL-1β: 25% | [38,52] |
| **SDF-1α** | Baseline during ischemia | EC | 75% | [32,53,54] |
| **Nitric Oxide** | WSS-induced | EC | WSS < 1 : 10%  1 < WSS < 2 : 20%  2 < WSS < 4 : 30%  WSS > 4 : 80% | [55,56] |
| **TGF-β** | Baseline | EC  Monocyte / MΦ | 20%  20% | [32,57] |
|  | Chemokine-induced | EC  Monocyte / MΦ | TGF-β: promote  TGF-β: promote | [58,59] |

**Table S2.** Chemokine activity as implemented within the ABM.

| **Adhesion Molecule** | **Activator** | **Responder** | **Stimulus: Probability** | **Citation** |
| --- | --- | --- | --- | --- |
| **LFA-1** | Chemokine-induced | Monocyte | MCP-1: 100% | [30,60] |
|  |  | hASC | MCP-1: 2% | Based on monocyte |
| **Mac-1** | Chemokine-induced | Monocyte | MCP-1: 100% | [30,60] |
|  |  | hASC | MCP-1: 43% | Based on monocyte |
| **CD54** | Δ in WSS-induced | EC | Increase: promote (75%) | [61,62] |
|  | Chemokine-induced | EC | IL-1β: 95% | [17,39,61-64] |
|  |  | EC | TNF−α: 90% | [17,63,65,66] |
|  |  | EC | TNF-α + + high WSS: 100% | [65] |
|  |  | Monocyte | TNF-α: 50% | [67] |
|  |  | hASC | TNF-α: 50% | Based on monocyte |
| **CD62E** | Chemokine-induced | EC | IL-1β: 95% | [17,61,62] |
|  |  | EC | TNF-α: 30% | [17,65,68,69] |
|  |  | EC | TNF-α + high WSS: 20% | [65] |
|  |  | EC | TGF-β: inhibit TNFα effects | [70] |
| **CD62P** | Chemokine-induced | EC | IL-1β: 30% | [71] |
| **CD106** | Δ in WSS-induced | EC | Increase: promote (30%) | [55,62,65,72-74] |
|  | Chemokine-induced | EC | IL-1β: 15% | [17,62,64,71] |
|  |  | EC | TNF-α: 60% | [17,39,69,71,73,74] |
|  |  | EC | NO + IL-1β: 5% | [39] |
|  |  | EC | NO + TNF-α: 30% | [39] |

**Table S3.** Chemokine- and WSS-induced changes in CAM as implemented in the ABM.

| **Adhesion Molecule(s)** | **Stimulus** | **Probability of Behavior** | **Citation** |
| --- | --- | --- | --- |
| **LFA-1** | WSS > 2 | A: 3% | [75] |
|  | MAC-1 + WSS > 2 | A: 8% | [75] |
|  | Ligated CD62L +  MAC-1 | A: 40% | [76] |
|  | IL-8 | A: 40% | [77,78] |
|  | SDF-1α | A: 90%  M: 12% | [79] |
| **VLA-4** | WSS < 2 | A: 30% | [80,81] |
|  | 2 < WSS < 4 | A: 22% | [80,81] |
|  | WSS > 4 | A: 10% | [80,81] |
|  | Ligated CD62P | A: 90% | [82] |
|  | SDF-1α | A: 45%  M: 22% | [79,83] |
| **MAC-1** | WSS > 2 | A: 5% | [75] |
|  | WSS > 2 + LFA-1 | A: 8% | [75] |
|  | Ligated CD62L +  LFA-1 | A: 50% | [76] |
|  | IL-8 | A: 20% | [77,78] |
| **Selectins (CD62E,P,L)** | WSS < 1  1 < WSS < 2.5  2.5 < WSS < 4  WSS > 4 | R: 25🡪50%  R: 50🡪90%  R: 5🡪50%  R: 0🡪5% | [25,39,73,84-89] |
| **CD62P** | TNFα | R: 2x (modifier) | [90] |
| **Integrins** | Activated | M: 3.5% | [79,91] |
|  | Activated + MCP-1 | M: 8.3% | [91] |
|  | Activated + MCP-1  + TNF-α | M: 3.5% | [91] |
|  | Activated + SDF-1α | M: 27% | [79] |
|  | All: WSS > 7  Monocytes: WSS < 7  hASCs: WSS > 5  hASCs: WSS > 3 | 100% detach  5% detach  50% detach  30% detach | [89,92-94] |
|  | IL-1β | A: 30% | [95] |

**Table S4.** Integrin activation as implemented in the ABM. Abbreviations are as follows: A = activation of integrins; M = probability of transmigration; R = probability of interaction (necessary for rolling to occur)

| **Group** | **Design** | | | | | **Metric** | | |
| --- | --- | --- | --- | --- | --- | --- | --- | --- |
|  | **Model** | **Cells** | **Time-points** | **Whole-body perfusion** | **Tracking** | **Incorporation** | **Limb health** | **Specificity** |
| Cao et al [96] | Nude mouse (8-10 weeks);  UHI | 1M (p=5);  i.v. at 2 hours | 14 days | Negative | RT-PCR for human EC markers; hindlimb sections | “incorporated into the new vessels” | Restoration of function; increased tissue perfusion (LDPI) | NA |
| Cai et al [97] | Nude mouse (8 weeks); UHI | 1M (p=4);  i.v. at 24 hours | 48 hours | Negative | GFP-labeled hASC; gastrocnemius sections | < 1%^*^ | Increased angiogenesis; increased tissue perfusion (LDPI) | 3.5x |
| Rehman et al [98] | Nude mouse (8 weeks); UHI | 500k (p=1);  i.v. at 24 hours | NA | NA | NA | NA | Restoration of function | NA |
| Miranville et al [15] | Nude mouse (8-10 weeks);  UHI | 500k (p=0);  i.v. at 24 hours | 14 days | Negative | HLA antibody; hindlimb sections | ~5% of vessels contained hASC | Increased capillary densitiy; increased tissue perfusion (LDPI) | NA |

**Table S5.** Literature review of select studies examining the therapeutic use of hASCs in the treatment of ischemic injury. In all, incorporation was either not assayed, subjective, or relied on tissue-sectioning. Time-points indicate when ischemic tissue was harvested and assessed for hASC incorporation, specifically, and does not include all assays or time-points used within each study. Whole-body perfusion was not performed prior to tissue harvesting in any of the studies, which is important for the identification of extra-vascular cells and assessment of hASC incorporation. Specificity was defined as the ratio of the number of incorporated cells in ischemic tissue versus the number in the contralateral control (healthy) tissue. Abbreviations are as follows: EC (endothelial cell); i.v. (intravenous delivery); LDPI (Laser Doppler perfusion imaging); NA (not applicable); UHI (unilateral hindlimb ischemia). ^*^Calculations based on assumptions of mouse gastrocnemius weight (0.14-0.266 g; [99]) and volume (90 + 10 mm^3^; [100]).

**Supplemental References**

1. Wilensky U (1999) NetLogo. In: University N, editor. Evanston, IL: Center for Connected Learning and Computer-based Modeling.

2. Bailey AM, Thorne BC, Peirce SM (2007) Multi-cell Agent-based Simulation of the Microvasculature to Study the Dynamics of Circulating Inflammatory Cell Trafficking. Ann Biomed Eng 35: 916-936.

3. Gronthos S, Franklin DM, Leddy HA, Robey PG, Storms RW, et al. (2001) Surface protein characterization of human adipose tissue-derived stromal cells. J Cell Physiol 189: 54-63.

4. Katz AJ, Tholpady A, Tholpady SS, Shang H, Ogle RC (2005) Cell surface and transcriptional characterization of human adipose-derived adherent stromal (hADAS) cells. Stem Cells 23: 412-423.

5. Elangbam CS, Qualls CW, Dahlgren RR (1997) Cell adhesion molecules--update. Vet Pathol 34: 61--73.

6. Pigott R, Power C (1993) The Adhesion Molecule Facts Book. New York: Harcourt Brace & Company, Publishers. 190 p.

7. Sbrana S, Bevilacqua S, Buffa M, Spiller D, Parri MS, et al. (2005) Post-reperfusion changes of monocyte function in coronary blood after extracorporeal circulation. Cytometry B Clin Cytom 65: 14-21.

8. Meyerrose TE, De Ugarte DA, Hofling AA, Herrbrich PE, Cordonnier TD, et al. (2007) In vivo distribution of human adipose-derived mesenchymal stem cells in novel xenotransplantation models. Stem Cells 25: 220-227.

9. Festy F, Hoareau L, Bes-Houtmann S, Pequin AM, Gonthier MP, et al. (2005) Surface protein expression between human adipose tissue-derived stromal cells and mature adipocytes. Histochem Cell Biol 124: 113-121.

10. Lee RH, Kim B, Choi I, Kim H, Choi HS, et al. (2004) Characterization and expression analysis of mesenchymal stem cells from human bone marrow and adipose tissue. Cell Physiol Biochem 14: 311-324.

11. Kern S, Eichler H, Stoeve J, Kluter H, Bieback K (2006) Comparative analysis of mesenchymal stem cells from bone marrow, umbilical cord blood, or adipose tissue. Stem Cells 24: 1294-1301.

12. Mitchell JB, McIntosh K, Zvonic S, Garrett S, Floyd ZE, et al. (2006) Immunophenotype of human adipose-derived cells: temporal changes in stromal-associated and stem cell-associated markers. Stem Cells 24: 376-385.

13. Hemler ME, Huang C, Takada Y, Schwarz L, Strominger JL, et al. (1987) Characterization of the cell surface heterodimer VLA-4 and related peptides. J Biol Chem 262: 11478--11485.

14. Hemler ME, Elices MJ, Parker C, Takada Y (1990) Structure of the integrin VLA-4 and its cell-cell and cell-matrix adhesion functions. Immunol Rev 114: 45-65.

15. Miranville A, Heeschen C, Sengenes C, Curat CA, Busse R, et al. (2004) Improvement of postnatal neovascularization by human adipose tissue-derived stem cells. Circulation 110: 349-355.

16. Wilhelmi MH, Leyh RG, Wilhelmi M, Haverich A (2005) Upregulation of endothelial adhesion molecules in hearts with congestive and ischemic cardiomyopathy: immunohistochemical evaluation of inflammatory endothelial cell activation. Eur J Cardiothorac Surg 27: 122-127.

17. Unger RE, Krump-Konvalinkova V, Peters K, Kirkpatrick CJ (2002) In vitro expression of the endothelial phenotype: comparative study of primary isolated cells and cell lines, including the novel cell line HPMEC-ST1.6R. Microvasc Res 64: 384-397.

18. Rothlein R, Dustin ML, Marlin SD, Springer TA (1986) A human intercellular adhesion molecule (ICAM-1) distinct from LFA-1. J Immunol 137: 1270-1274.

19. Dustin ML, Rothlein R, Bhan AK, Dinarello CA, Springer TA (1986) Induction by IL 1 and interferon-gamma: tissue distribution, biochemistry, and function of a natural adherence molecule (ICAM-1). J Immunol 137: 245-254.

20. Fox JM, Chamberlain G, Ashton BA, Middleton J (2007) Recent advances into the understanding of mesenchymal stem cell trafficking. Br J Haematol 137: 491-502.

21. Jackson LA, Drevets DA, Dong ZM, Greenfield RA, Murphy JW (2005) Levels of L-selectin (CD62L) on human leukocytes in disseminated cryptococcosis with and without associated HIV-1 infection. J Infect Dis 191: 1361-1367.

22. Shimada Y, Hasegawa M, Takehara K, Sato S (2001) Elevated serum L-selectin levels and decreased L-selectin expression on CD8(+) lymphocytes in systemic sclerosis. Clin Exp Immunol 124: 474-479.

23. Sako D, Chang XJ, Barone KM, Vachino G, White HM, et al. (1993) Expression cloning of a functional glycoprotein ligand for P-selectin. Cell 75: 1179--1186.

24. Chukwuemeka AO, Brown KA, Venn GE, Chambers DJ (2005) Changes in P-selectin expression on cardiac microvessels in blood-perfused rat hearts subjected to ischemia-reperfusion. Ann Thorac Surg 79: 204-211.

25. Kim MB, Sarelius IH (2004) Role of shear forces and adhesion molecule distribution on P-selectin-mediated leukocyte rolling in postcapillary venules. Am J Physiol Heart Circ Physiol 287: H2705-2711.

26. Stohlawetz P, Hahn P, Koller M, Hauer J, Resch H, et al. (1998) Immunophenotypic characteristics of monocytes in elderly subjects. Scand J Immunol 48: 324-326.

27. Moore KL, Patel KD, Bruehl RE, Li F, Johnson DA, et al. (1995) P-selectin glycoprotein ligand-1 mediates rolling of human neutrophils on P-selectin. J Cell Biol 128: 661--671.

28. da Costa Martins P, Garcia-Vallejo JJ, van Thienen JV, Fernandez-Borja M, van Gils JM, et al. (2007) P-selectin glycoprotein ligand-1 is expressed on endothelial cells and mediates monocyte adhesion to activated endothelium. Arterioscler Thromb Vasc Biol 27: 1023-1029.

29. Dinarello CA, Ikejima T, Warner SJ, Orencole SF, Lonnemann G, et al. (1987) Interleukin 1 induces interleukin 1. I. Induction of circulating interleukin 1 in rabbits in vivo and in human mononuclear cells in vitro. J Immunol 139: 1902-1910.

30. Jiang Y, Beller DI, Frendl G, Graves DT (1992) Monocyte chemoattractant protein-1 regulates adhesion molecule expression and cytokine production in human monocytes. J Immunol 148: 2423-2428.

31. Kern S, Robertson SA, Mau VJ, Maddocks S (1995) Cytokine secretion by macrophages in the rat testis. Biol Reprod 53: 1407-1416.

32. Tedgui A, Mallat Z (2006) Cytokines in atherosclerosis: pathogenic and regulatory pathways. Physiol Rev 86: 515-581.

33. van der Meer JW, Endres S, Lonnemann G, Cannon JG, Ikejima T, et al. (1988) Concentrations of immunoreactive human tumor necrosis factor alpha produced by human mononuclear cells in vitro. J Leukoc Biol 43: 216-223.

34. Bogdan C, Nathan C (1993) Modulation of macrophage function by transforming growth factor beta, interleukin-4, and interleukin-10. Ann N Y Acad Sci 685: 713-739.

35. Libby P, Ordovas JM, Auger KR, Robbins AH, Birinyi LK, et al. (1986) Endotoxin and tumor necrosis factor induce interleukin-1 gene expression in adult human vascular endothelial cells. Am J Pathol 124: 179-185.

36. Locksley RM, Heinzel FP, Shepard HM, Agosti J, Eessalu TE, et al. (1987) Tumor necrosis factors alpha and beta differ in their capacities to generate interleukin 1 release from human endothelial cells. J Immunol 139: 1891-1895.

37. Jiang Z, Berceli SA, Pfahnl CL, Wu L, Goldman D, et al. (2004) Wall shear modulation of cytokines in early vein grafts. J Vasc Surg 40: 345-350.

38. Lukacs NW, Strieter RM, Elner V, Evanoff HL, Burdick MD, et al. (1995) Production of chemokines, interleukin-8 and monocyte chemoattractant protein-1, during monocyte: endothelial cell interactions. Blood 86: 2767-2773.

39. De Caterina R, Libby P, Peng HB, Thannickal VJ, Rajavashisth TB, et al. (1995) Nitric oxide decreases cytokine-induced endothelial activation. Nitric oxide selectively reduces endothelial expression of adhesion molecules and proinflammatory cytokines. J Clin Invest 96: 60-68.

40. DeForge LE, Kenney JS, Jones ML, Warren JS, Remick DG (1992) Biphasic production of IL-8 in lipopolysaccharide (LPS)-stimulated human whole blood. Separation of LPS- and cytokine-stimulated components using anti-tumor necrosis factor and anti-IL-1 antibodies. J Immunol 148: 2133-2141.

41. Hwang YS, Jeong M, Park JS, Kim MH, Lee DB, et al. (2004) Interleukin-1beta stimulates IL-8 expression through MAP kinase and ROS signaling in human gastric carcinoma cells. Oncogene 23: 6603-6611.

42. Chen CC, Manning AM (1996) TGF-beta 1, IL-10 and IL-4 differentially modulate the cytokine-induced expression of IL-6 and IL-8 in human endothelial cells. Cytokine 8: 58-65.

43. Liang F, Huang N, Wang B, Chen H, Wu L (2002) Shear stress induces interleukin-8 mRNA expression and transcriptional activation in human vascular endothelial cells. Chin Med J (Engl) 115: 1838-1842.

44. Tang R, Cheng M, Nie Y, Chen H (2004) [Force-dependent effects of interleukin-8 production in endothelial cells exposed to fluid shear stress]. Sheng Wu Yi Xue Gong Cheng Xue Za Zhi 21: 363-366.

45. Daftarian PM, Kumar A, Kryworuchko M, Diaz-Mitoma F (1996) IL-10 production is enhanced in human T cells by IL-12 and IL-6 and in monocytes by tumor necrosis factor-alpha. J Immunol 157: 12-20.

46. Wanidworanun C, Strober W (1993) Predominant role of tumor necrosis factor-alpha in human monocyte IL-10 synthesis. J Immunol 151: 6853-6861.

47. Maeda H, Kuwahara H, Ichimura Y, Ohtsuki M, Kurakata S, et al. (1995) TGF-beta enhances macrophage ability to produce IL-10 in normal and tumor-bearing mice. J Immunol 155: 4926-4932.

48. Bogdan C, Vodovotz Y, Nathan C (1991) Macrophage deactivation by interleukin 10. J Exp Med 174: 1549-1555.

49. Weiss JM, Cuff CA, Berman JW (1999) TGF-beta downmodulates cytokine-induced monocyte chemoattractant protein (MCP)-1 expression in human endothelial cells. A putative role for TGF-beta in the modulation of TNF receptor expression. Endothelium 6: 291-302.

50. Xiao YQ, Malcolm K, Worthen GS, Gardai S, Schiemann WP, et al. (2002) Cross-talk between ERK and p38 MAPK mediates selective suppression of pro-inflammatory cytokines by transforming growth factor-beta. J Biol Chem 277: 14884-14893.

51. Yu H, Zeng Y, Hu J, Li C (2002) Fluid shear stress induces the secretion of monocyte chemoattractant protein-1 in cultured human umbilical vein endothelial cells. Clin Hemorheol Microcirc 26: 199-207.

52. Takahashi M, Masuyama J, Ikeda U, Kasahara T, Kitagawa S, et al. (1995) Induction of monocyte chemoattractant protein-1 synthesis in human monocytes during transendothelial migration in vitro. Circ Res 76: 750-757.

53. Ceradini DJ, Kulkarni AR, Callaghan MJ, Tepper OM, Bastidas N, et al. (2004) Progenitor cell trafficking is regulated by hypoxic gradients through HIF-1 induction of SDF-1. Nat Med 10: 858-864.

54. Salvucci O, Yao L, Villalba S, Sajewicz A, Pittaluga S, et al. (2002) Regulation of endothelial cell branching morphogenesis by endogenous chemokine stromal-derived factor-1. Blood 99: 2703-2711.

55. Nerem RM, Alexander RW, Chappell DC, Medford RM, Varner SE, et al. (1998) The study of the influence of flow on vascular endothelial biology. Am J Med Sci 316: 169-175.

56. Luscher TF (1991) Endothelium-derived nitric oxide: the endogenous nitrovasodilator in the human cardiovascular system. Eur Heart J 12 Suppl E: 2-11.

57. Ross R (1993) The pathogenesis of atherosclerosis: a perspective for the 1990s. Nature 362: 801-809.

58. Kim SJ, Angel P, Lafyatis R, Hattori K, Kim KY, et al. (1990) Autoinduction of transforming growth factor beta 1 is mediated by the AP-1 complex. Mol Cell Biol 10: 1492-1497.

59. Singh NN, Ramji DP (2006) The role of transforming growth factor-beta in atherosclerosis. Cytokine Growth Factor Rev 17: 487-499.

60. Vaddi K, Newton RC (1994) Regulation of monocyte integrin expression by beta-family chemokines. J Immunol 153: 4721-4732.

61. Morigi M, Zoja C, Figliuzzi M, Foppolo M, Micheletti G, et al. (1995) Fluid shear stress modulates surface expression of adhesion molecules by endothelial cells. Blood 85: 1696-1703.

62. Nagel T, Resnick N, Atkinson WJ, Dewey CF, Jr., Gimbrone MA, Jr. (1994) Shear stress selectively upregulates intercellular adhesion molecule-1 expression in cultured human vascular endothelial cells. J Clin Invest 94: 885-891.

63. Haraldsen G, Kvale D, Lien B, Farstad IN, Brandtzaeg P (1996) Cytokine-regulated expression of E-selectin, intercellular adhesion molecule-1 (ICAM-1), and vascular cell adhesion molecule-1 (VCAM-1) in human microvascular endothelial cells. J Immunol 156: 2558-2565.

64. Chappell DC, Varner SE, Nerem RM, Medford RM, Alexander RW (1998) Oscillatory shear stress stimulates adhesion molecule expression in cultured human endothelium. Circ Res 82: 532-539.

65. Chiu JJ, Lee PL, Chen CN, Lee CI, Chang SF, et al. (2004) Shear stress increases ICAM-1 and decreases VCAM-1 and E-selectin expressions induced by tumor necrosis factor-[alpha] in endothelial cells. Arterioscler Thromb Vasc Biol 24: 73-79.

66. Swerlick RA, Lee KH, Li LJ, Sepp NT, Caughman SW, et al. (1992) Regulation of vascular cell adhesion molecule 1 on human dermal microvascular endothelial cells. J Immunol 149: 698-705.

67. Yoshida A, Takahashi HK, Nishibori M, Iwagaki H, Yoshino T, et al. (2001) IL-18-induced expression of intercellular adhesion molecule-1 in human monocytes: involvement in IL-12 and IFN-gamma production in PBMC. Cell Immunol 210: 106-115.

68. Aziz KE, Wakefield D (1996) Modulation of endothelial cell expression of ICAM-1, E-selectin, and VCAM-1 by beta-estradiol, progesterone, and dexamethasone. Cell Immunol 167: 79-85.

69. Briscoe DM, Cotran RS, Pober JS (1992) Effects of tumor necrosis factor, lipopolysaccharide, and IL-4 on the expression of vascular cell adhesion molecule-1 in vivo. Correlation with CD3+ T cell infiltration. J Immunol 149: 2954-2960.

70. DiChiara MR, Kiely JM, Gimbrone MA, Jr., Lee ME, Perrella MA, et al. (2000) Inhibition of E-selectin gene expression by transforming growth factor beta in endothelial cells involves coactivator integration of Smad and nuclear factor kappaB-mediated signals. J Exp Med 192: 695-704.

71. Abe Y, Ballantyne CM, Smith CW (1996) Functions of domain 1 and 4 of vascular cell adhesion molecule-1 in alpha4 integrin-dependent adhesion under static and flow conditions are differentially regulated. J Immunol 157: 5061-5069.

72. Ando J, Tsuboi H, Korenaga R, Takada Y, Toyama-Sorimachi N, et al. (1994) Shear stress inhibits adhesion of cultured mouse endothelial cells to lymphocytes by downregulating VCAM-1 expression. Am J Physiol 267: C679-687.

73. Gonzales RS, Wick TM (1996) Hemodynamic modulation of monocytic cell adherence to vascular endothelium. Ann Biomed Eng 24: 382-393.

74. Yamawaki H, Lehoux S, Berk BC (2003) Chronic physiological shear stress inhibits tumor necrosis factor-induced proinflammatory responses in rabbit aorta perfused ex vivo. Circulation 108: 1619-1625.

75. Neelamegham S, Taylor AD, Burns AR, Smith CW, Simon SI (1998) Hydrodynamic shear shows distinct roles for LFA-1 and Mac-1 in neutrophil adhesion to intercellular adhesion molecule-1. Blood 92: 1626-1638.

76. Kukreti S, Konstantopoulos K, Smith CW, McIntire LV (1997) Molecular mechanisms of monocyte adhesion to interleukin-1beta-stimulated endothelial cells under physiologic flow conditions. Blood 89: 4104-4111.

77. Gopalan PK, Smith CW, Lu H, Berg EL, McIntire LV, et al. (1997) Neutrophil CD18-dependent arrest on intercellular adhesion molecule 1 (ICAM-1) in shear flow can be activated through L-selectin. J Immunol 158: 367-375.

78. Lum AF, Green CE, Lee GR, Staunton DE, Simon SI (2002) Dynamic regulation of LFA-1 activation and neutrophil arrest on intercellular adhesion molecule 1 (ICAM-1) in shear flow. J Biol Chem 277: 20660-20670.

79. Peled A, Kollet O, Ponomaryov T, Petit I, Franitza S, et al. (2000) The chemokine SDF-1 activates the integrins LFA-1, VLA-4, and VLA-5 on immature human CD34(+) cells: role in transendothelial/stromal migration and engraftment of NOD/SCID mice. Blood 95: 3289-3296.

80. Zwartz GJ, Chigaev A, Dwyer DC, Foutz TD, Edwards BS, et al. (2004) Real-time analysis of very late antigen-4 affinity modulation by shear. J Biol Chem 279: 38277-38286.

81. Chigaev A, Zwartz G, Graves SW, Dwyer DC, Tsuji H, et al. (2003) Alpha4beta1 integrin affinity changes govern cell adhesion. J Biol Chem 278: 38174-38182.

82. Yago T, Tsukuda M, Minami M (1999) P-selectin binding promotes the adhesion of monocytes to VCAM-1 under flow conditions. J Immunol 163: 367-373.

83. Chan JR, Hyduk SJ, Cybulsky MI (2001) Chemoattractants induce a rapid and transient upregulation of monocyte alpha4 integrin affinity for vascular cell adhesion molecule 1 which mediates arrest: an early step in the process of emigration. J Exp Med 193: 1149-1158.

84. Alon R, Chen S, Fuhlbrigge R, Puri KD, Springer TA (1998) The kinetics and shear threshold of transient and rolling interactions of L-selectin with its ligand on leukocytes. Proc Natl Acad Sci U S A 95: 11631-11636.

85. Chen S, Alon R, Fuhlbrigge RC, Springer TA (1997) Rolling and transient tethering of leukocytes on antibodies reveal specializations of selectins. Proc Natl Acad Sci U S A 94: 3172-3177.

86. Lawrence MB, Springer TA (1993) Neutrophils roll on E-selectin. J Immunol 151: 6338-6346.

87. Sarangapani KK, Yago T, Klopocki AG, Lawrence MB, Fieger CB, et al. (2004) Low force decelerates L-selectin dissociation from P-selectin glycoprotein ligand-1 and endoglycan. J Biol Chem 279: 2291-2298.

88. Yago T, Wu J, Wey CD, Klopocki AG, Zhu C, et al. (2004) Catch bonds govern adhesion through L-selectin at threshold shear. J Cell Biol 166: 913-923.

89. Peled A, Grabovsky V, Habler L, Sandbank J, Arenzana-Seisdedos F, et al. (1999) The chemokine SDF-1 stimulates integrin-mediated arrest of CD34(+) cells on vascular endothelium under shear flow. J Clin Invest 104: 1199-1211.

90. Kunkel EJ, Jung U, Ley K (1997) TNF-alpha induces selectin-mediated leukocyte rolling in mouse cremaster muscle arterioles. Am J Physiol 272: H1391-1400.

91. Weber C, Draude G, Weber KS, Wubert J, Lorenz RL, et al. (1999) Downregulation by tumor necrosis factor-alpha of monocyte CCR2 expression and monocyte chemotactic protein-1-induced transendothelial migration is antagonized by oxidized low-density lipoprotein: a potential mechanism of monocyte retention in atherosclerotic lesions. Atherosclerosis 145: 115-123.

92. Rinker KD, Prabhakar V, Truskey GA (2001) Effect of contact time and force on monocyte adhesion to vascular endothelium. Biophys J 80: 1722-1732.

93. Greenberg AW, Kerr WG, Hammer DA (2000) Relationship between selectin-mediated rolling of hematopoietic stem and progenitor cells and progression in hematopoietic development. Blood 95: 478-486.

94. Ruster B, Gottig S, Ludwig RJ, Bistrian R, Muller S, et al. (2006) Mesenchymal stem cells display coordinated rolling and adhesion behavior on endothelial cells. Blood 108: 3938-3944.

95. Bevilacqua MP, Pober JS, Wheeler ME, Cotran RS, Gimbrone MA, Jr. (1985) Interleukin 1 acts on cultured human vascular endothelium to increase the adhesion of polymorphonuclear leukocytes, monocytes, and related leukocyte cell lines. J Clin Invest 76: 2003-2011.

96. Cao Y, Sun Z, Liao L, Meng Y, Han Q, et al. (2005) Human adipose tissue-derived stem cells differentiate into endothelial cells in vitro and improve postnatal neovascularization in vivo. Biochem Biophys Res Commun 332: 370-379.

97. Cai L, Johnstone BH, Cook TG, Liang Z, Traktuev D, et al. (2007) Suppression of hepatocyte growth factor production impairs the ability of adipose-derived stem cells to promote ischemic tissue revascularization. Stem Cells 25: 3234-3243.

98. Rehman J, Traktuev D, Li J, Merfeld-Clauss S, Temm-Grove CJ, et al. (2004) Secretion of angiogenic and antiapoptotic factors by human adipose stromal cells. Circulation 109: 1292-1298.

99. Langen RC, Schols AM, Kelders MC, van der Velden JL, Wouters EF, et al. (2006) Muscle wasting and impaired muscle regeneration in a murine model of chronic pulmonary inflammation. Am J Respir Cell Mol Biol 35: 689-696.

100. Zhang J, Zhang G, Morrison B, Mori S, Sheikh KA (2008) Magnetic resonance imaging of mouse skeletal muscle to measure denervation atrophy. Exp Neurol 212: 448-457.
